# The first checklist of alien vascular plants of Kyrgyzstan, with new records and critical evaluation of earlier data. Contribution 3

**DOI:** 10.3897/BDJ.13.e145624

**Published:** 2025-03-04

**Authors:** Alexander Sennikov, Georgy Lazkov, Dmitry A. German

**Affiliations:** 1 University of Helsinki, Helsinki, Finland University of Helsinki Helsinki Finland; 2 Institute of Biology, Bishkek, Kyrgyzstan Institute of Biology Bishkek Kyrgyzstan; 3 Altai State University, Barnaul, Russia Altai State University Barnaul Russia

**Keywords:** Brassicaceae, casual aliens, Central Asia, established aliens, introduction, naturalisation, non-native plants, plant invasions

## Abstract

**Background:**

We continue the series of detailed treatments of alien vascular plants of Kyrgyzstan. The complete background for every species occurrence (herbarium specimens, documented observations, published literature) is uncovered and critically evaluated in a wide context of plant invasions in Central Asia with a reference to Eastern Europe and Northern Asia, based on events in the political and economic history. Complete point distribution maps are provided for each species in Central Asia, in general and Kyrgyzstan, in particular.

**New information:**

All records of *Hesperismatronalis* in Central Asia (including Kyrgyzstan) belong to *H.pycnotricha*; the latter species is newly reported as a locally naturalised alien in Kazakhstan. The previous record of *Sisymbriumirio* from Kyrgyzstan is rejected as based on a misidentified specimen of *S.loeselii*, but the species is newly recorded here as a recent casual alien. *Hirschfeldiaincana* is presumably native in south-western Turkmenistan; its second record in Central Asia was caused by the import of contaminated wheat grain in the times of the Soviet grain crisis and its recent expansion may be linked to the increasing import of forage grain. The introduction of *Crambeorientalis* was connected with its cultivation for fodder and as an ornamental plant and its further broad dispersal was aided by winds. *Rorippaaustriaca* is native in the steppes of north-western Kazakhstan, but alien in the mountains of Central Asia. The occurrences of three alien species originated directly from cultivation (*Hesperispycnotricha* as an ornamental, *Armoraciarusticana* as an edible plant, *Crambeorientalis* as an ornamental and fodder plant), three species (*Hirschfeldiaincana*, *Mutardaarvensis*, *Sisymbriumirio*) were imported as grain contaminants, whereas two others (*Rorippaaustriaca*, *R.sylvestris*) have arrived with contaminated soil on ornamental plants or arboreous saplings. The arrival period is inferred as the Neolithic period (*Mutardaarvensis*), the Imperial times (*Armoraciarusticana*, *Hesperispycnotricha*), the post-war Soviet times (*Crambeorientalis*, *Rorippaaustriaca*, *R.sylvestris*) and the independence times (*Hirschfeldiaincana*, *Sisymbriumirio*). All the treated species, but two, increase their frequency in Kyrgyzstan; *Mutardaarvensis* has already reached its complete distribution, being an ubiquitous weed, whereas *Armoraciarusticana* experiences a projected decline because its common cultivation has ceased. No species is invasive in natural habitats. A new combination, Mutardaarvensisvar.orientalis (L.) Sennikov, is proposed for a variant with pubescent pods.

## Introduction

The spread of invasive alien species is a major aspect of negative human impact, which is cited as a matter of global concern that requires urgent actions from scientists, decision-makers and the society (Pyšek et al. 2020). The negative impact of alien species is manifold, not only reducing the agricultural production and affecting the human well-being and the cultural value of landscapes (Shackleton et al. 2018), but also causing extensive damage to natural ecosystems (Pejchar and Mooney 2009). For practical reasons, causing economic or environmental harm is included in the U.S. Government definition of invasive species (Reaser et al. 2020).

The data representation from Central Asia is highly deficient in global databases (Ondo et al. 2024), despite a long tradition of botanical research with an extensive legacy of high-quality taxonomic and floristic treatments (Sennikov et al. 2016). To contribute for overcoming this deficiency and to respond to the recent calls for action and development of invasive species management (e.g. Speziale and Lambertucci (2010), Colberg et al. (2024)), a comprehensive inventory of the taxonomic composition, geographical distribution and pathways of introduction of alien plants has been initiated for Kyrgyzstan (Sennikov and Lazkov 2021, Sennikov and Lazkov 2022). As an important step towards the inventory and understanding of the harmful impact of alien plants in the country, their first complete checklist has been published (Sennikov and Lazkov 2024a). The updatable version of this checklist (Sennikov and Lazkov 2024c) supersedes the earlier effort made for the Global Register of Introduced and Invasive Species (Sennikov et al. 2021).

According to Sennikov and Lazkov (2024a), 151 alien plant species have been registered in Kyrgyzstan, of which nearly 40% are believed to have become naturalised at least locally. Mediterranean plants were found to dominate in the list, but their actual source, period and pathways of introduction have not been uncovered at that time because of the obscurity of the primary data in the background. As human history is a major factor shaping the global distribution of alien plants (Fristoe et al. 2023), its connection with the distribution of alien plants in Central Asia is the main subject of the present series of research papers.

Brassicaceae is a large family of vascular plants, including some 4000 currently accepted species, about 350 genera and 58 tribes; its phylogenetic tree demonstrates an extensive radiation promoted by rampant hybridisation among closely and more distantly related lineages (Hendriks et al. 2023). The family is of major economic and cultural importance for mankind, with numerous representatives having been grown since prehistoric times, as edible, fodder, oilseed, biofuel and ornamental plants (Appel and Al-Shehbaz 2003, Warwick 2011). Many species of Brassicaceae occur in arid or semi-arid environments, thus being well represented as native to Central Asia and being capable to naturalise in its arid climate. The potential ease of their human-mediated transfer is indicated already by numerous disjunct distributions in Brassicales, which are either ancient or more recent (Fay and Christenhusz 2010). Indeed, Brassicaceae are notorious for their numerous invasive representatives; in Europe, this family concludes the top-five with the greatest representation of alien taxa (Pyšek et al. 2009). The species of Brassicaceae may possess diverse traits that facilitate their potential invasion, which may need to be evaluated in each case separately (Hurka et al. 2003).

Brassicaceae is the second leading family of alien vascular plants in Kyrgyzstan, among other taxonomic groups that are typical of arid zones; at the same time, this family is characterised by the lowest level of naturalisation in the country. Sixteen alien species of Brassicaceae have been registered in Kyrgyzstan (Sennikov and Lazkov 2024a). In the present contribution, we collected the occurrence data and the relevant literature for half of this species set, in order to uncover their historical and actual distribution and to link their appearance in the territory with the major human activities. To provide a broader context and more precise and reliable interpretation of the data from Kyrgyzstan, we collected and interpreted comprehensive and detailed information for the whole territory of Central Asia.

## Materials and methods

The checklist contains taxonomic and distributional information on selected species of the Brassicaceae family. The species list is alphabetically organised (according to genera and species) and the content is structured according to Sennikov and Lazkov (2021). A strong emphasis is placed on the time and pathways of introduction and the current status and impact of certain species in Kyrgyzstan, in the context of plant invasions in Central Asia as a whole.

Species records in Kyrgyzstan, in particular and Central Asia, in general were traced comprehensively on the basis of herbarium collections and documented observations, to which the authors' field observations have been supplemented. The collections of main Herbaria, in which recent and historical specimens from Central Asia are known to have been deposited (AA, FRU, LE, MW, P, TASH), were screened and the taxonomic identity of each specimen was re-evaluated. The specimens were examined mostly *de visu*, but partly as digital images. Personal herbarium collections were deposited to ALTB (D. German), FRU (G. Lazkov) and H (A. Sennikov). Further records were traced from documented observations published through citizen-science platforms (iNaturalist 2024, Plantarium 2024); however, we refrained from automated harvesting of undocumented observations available through iNaturalist and GBIF (GBIF 2024) due to a high level of misidentifications. Distributional data for Tajikistan and Turkmenistan were complemented from published literature (Vasilczenko 1948, Ovchinnikov 1978). When original georeferences were not available, records were georeferenced *ad hoc* using contemporary and modern topographic maps, published gazetteers and datasets. Transcripts of the geographic data in Kyrgyzstan were standardised according to Ömürzakov et al. (1988) and transliterated from Cyrillic to Latin script; modern official transliterations were used for Kazakh and Uzbek languages.

The occurrences used in the present contribution are available as Suppl. material 1 (Kazakhstan, Tajikistan, Turkmenistan, Uzbekistan) and Suppl. material 2 (Kyrgyzstan). Distributional maps were created on the basis of these records in QGIS for Windows (QGIS 2024).

The information on species distributions outside Central Asia was derived from POWO (2024) and various taxonomic and floristic accounts, of which most important are Flora of Siberia (Malyschev and Peschkova 1994), Flora Iranica (Rechinger 1968), Flora of West Pakistan (Jafri 1973) and Flora of Turkey and the East Aegean Islands (Davis 1965).

The pathways of introduction are formalised according to Hulme et al. (2008) and Harrower et al. (2018). The invasion status is assessed according to Richardson et al. (2001) and Pyšek et al. (2004). Species dynamics are observed or inferred from the past (50-150 years ago) and current (the latest 20 years) distributional data, and expressed as decreasing, stable or increasing without quantification.

The taxonomy and nomenclature are based on the authors' assessments and may deviate from those in the public taxonomic and nomenclatural databases (IPNI 2024, POWO 2024).

## Data resources

### Taxonomic checklist

The present contribution builds on the taxonomic inventory of alien vascular plants of Kyrgyzstan (Sennikov and Lazkov 2024a), which is available as a dynamic checklist dataset in GBIF (Sennikov and Lazkov 2024c). This dataset also incorporates the taxonomic additions and corrections published in the present contribution.

### Occurrence dataset

The primary data for the distributional records published in the present contribution have been databased in the DarwinCore format and incorporated in the occurrence dataset of alien vascular plants of Kyrgyzstan, which is available through GBIF (Sennikov and Lazkov 2024b).

## Taxon treatments

### Armoracia rusticana

G.Gaertn., B.Mey. & Scherb., 1800

9A5584A8-AEB1-5F57-B0E2-D1735344DD6D

urn:lsid:ipni.org:names:278747-1

***Armoraciarusticana*** G.Gaertn., B.Mey. & Scherb., Oekon. Fl. Wetterau 2: 426 (1800) — *Cochleariaarmoracia* L., Sp. Pl. 2: 648 (1753).

#### Distribution

##### Native distribution

Cultigenic.

Although some earlier studies indicated that the species is native to Eastern Europe (Candolle 1908, Al-Shehbaz 1988), its natural populations are lacking (Kotov 1979, Ball 1993, Sampliner and Miller 2009).

##### Secondary distribution

Europe, temperate and northern Asia, temperate and northern North America.

The species may persist in places of its original cultivation for an uncertainly long time (Wedelsbäck Bladh et al. 2014). Its secondary distribution seems to be exclusively linked with cultivation places. Further dispersal occurs with soil transportation in populated places and along roads and with water currents along rivers.

In Europe, *Armoraciarusticana* is very common in the territories of its former cultivation (Jalas and Suominen 1994). In comprehensive recording activities, as for example, in Lithuania (Gudžinskas and Rašomavičius 2023), the species may be found commonly naturalised and persisting in the territory, with an active secondary dispersal into nature with garden waste and subsequent soil disturbance by road construction and maintenance.

In Northern Asia, the species has been registered as alien in nearly all floristic regions (Chepinoga et al. 2024), whereas its occurrence in China is limited to five provinces (Zhou et al. 2001), in which the species was first found only at the beginning of the 20^th^ century (Xu et al. 2012).

Up to the mid-20^th^ century, most of the sources indicated that *Armoraciarusticana* is a cultivated plant that becomes ruderal at the places of its cultivation. In Siberia, the species was cultivated at least from the second half of the 19^th^ century (Ankipovich and Ebel 2016), apparently introduced in the course of extensive colonisation from the central and then southern European parts of the Russian Empire (Vinogradova 2016) and considered restricted to anthropogenic landscapes by the first quarter of the 20^th^ century (Krylov 1931). In the 21^st^ century, numerous regional reports have noted the dispersal and naturalisation of the species into natural habitats along watercourses (e.g. Zykova (2023)). Currently, the species is listed among invasive vascular plants in Siberia (Ankipovich and Ebel 2016).

##### Distribution in Central Asia

**Figure 1.**
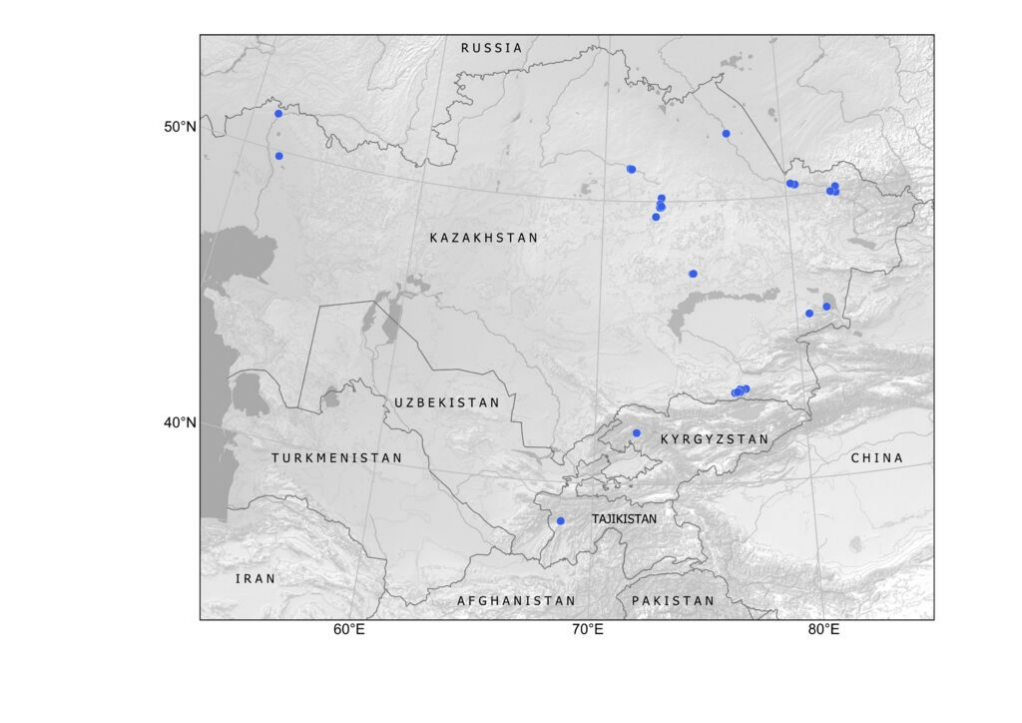
Subspontaneous records of *Armoraciarusticana* in Central Asia, according to herbarium specimens and documented observations (iNaturalist 2024, Plantarium 2024).

Kazakhstan, Kyrgyzstan, Tajikistan, Uzbekistan. Available subspontaneous records are shown in Fig. 1.

**Figure 2.**
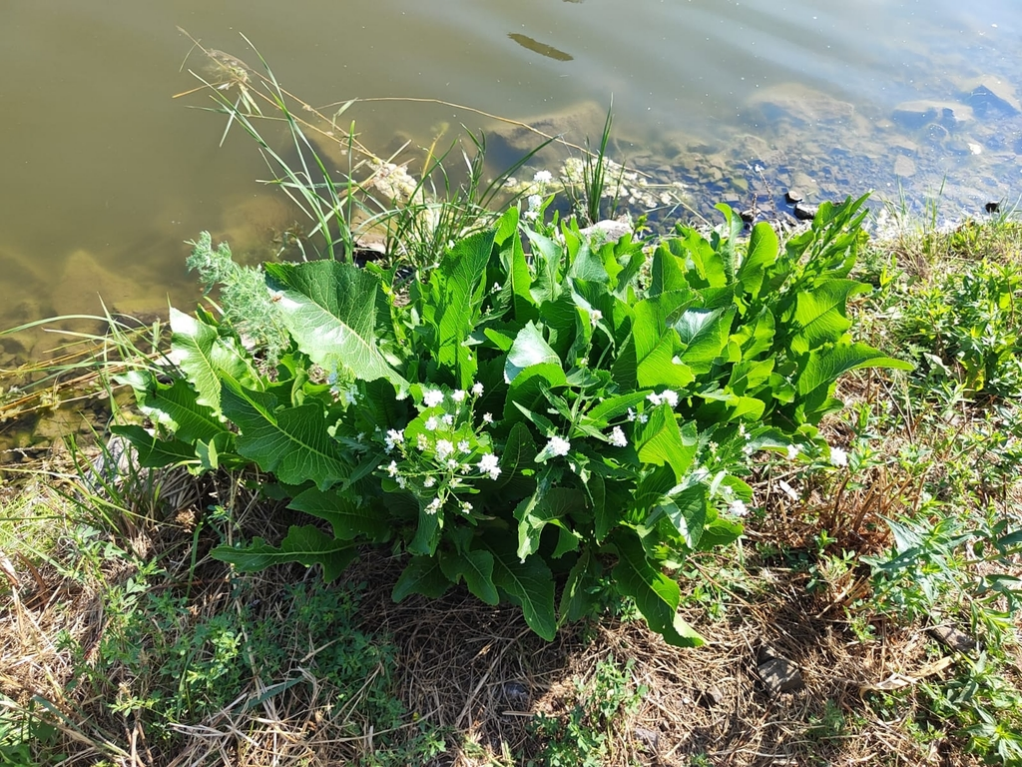
Naturalised stands of *Armoraciarusticana* along a bridge across the Ishim River, Astana City (photo by Yu. Morozova, 1 June 2023). Source: https://www.inaturalist.org/observations/166388104 (Plantarium 2024).

In Kazakhstan, *Armoraciarusticana* was cultivated in private vegetable gardens by Russian colonists, who brought this highly popular condiment from Central Russia (Gubanov et al. 1976). According to herbarium records, the species was known in cultivation in Almaty by 1886 and found naturalised in native habitats along rivers in the north-western regions in the 1920s and in the north-eastern regions by 1960 (Vasilieva 1961), where it was certainly present for a significant period of time before being recorded. Further reports of alien occurrence are known from the northern regions (Perezhogin et al. 2023). Recent records (Fig. 2) indicate its extensive spread along rivers around large towns of the north-east, for example, Semei (formerly Semipalatinsk), Öskemen (formerly Ust-Kamenogorsk) (Plantarium 2024) and Astana (iNaturalist 2024). Numerous recent records from the southern regions (iNaturalist 2024) are from mostly ruderal populations and belong to the dispersal from Soviet-time garden cultivation.

In Uzbekistan, the species has been known from cultivation (Botschantzev and Vvedensky 1955). Currently, it remains in use not only in larger towns, but also in Uzbek villages in the mountainous regions (Kosimov et al. 2023). So far, its running wild has not been recorded in the country (Sennikov et al. 2020).

In Kyrgyzstan, Nikitina (1955) considered the species as present only in cultivation. Its alien occurrence has been reported by Deza (1989) without any further particulars and taken into account in the recent compilations (Lazkov and Sultanova 2011, Lazkov and Sultanova 2014, Sennikov and Lazkov 2024a).

The latest account for Tajikistan (Yunusov 1978) reported the species as cultivated and sometimes escaping from cultivation, without further details on the alien status. No information on any particular subspontaneous occurrence in the country has been found. Nowak et al. (2020) claimed the species status of archaeophyte in the country, but the status is clearly in error.

##### Distribution in Kyrgyzstan

**Figure 3.**
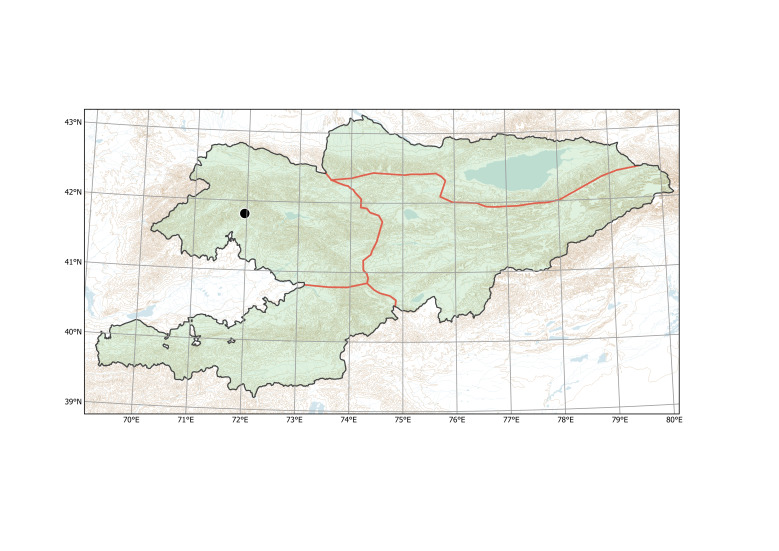
The confirmed alien record of *Armoraciarusticana* in Kyrgyzstan.

Western Tian-Shan (Fig. 3).

The background for the information in Deza (1989) is uncertain and may be an assumption or extrapolation. So far, we have not observed the species in ruderal localities in urban areas. The subspontaneous presence of the species in Kyrgyzstan is supported by our observation of its occurrence in a single locality in the Sary-Chelek Nature Reserve, where it grows on a roadside meadow immediately north of Arkyt Village.

#### Ecology

The native distribution of *Armoraciarusticana* is lacking. Its closest relative, *A.macrocarpa* (Waldst. & Kit. ex Willd.) Kit. ex Baumg., occurs on alluvial meadows and margins of wetlands in floodplains (Ball 1993).

In the secondary distribution area, the species can be found in a variety of ruderal places, along roadsides, on disturbed or anthropogenous meadows, along ditches and riversides. It may become invasive by colonising river valleys (Pyšek et al. 2012).

#### Biology

Taproot perennial. Strong thickened roots of *Armoraciarusticana* act as rhizomes because of their capability to produce new shoots and offsprings from their smallest fraction (Davis 1893). This feature is used in propagation of the plants in cultivation, and facilitates their secondary dispersal by soil transportation, which is especially common in Northern Europe (Gudžinskas and Rašomavičius 2023).

#### Taxon discussion

*Armoraciarusticana* is very close to *A.macrocarpa*. The latter species is a Pannonian endemic, restricted to the Danube Basin, which differs from *A.rusticana* primarily by its longer siliculae (10-15 mm vs. 4-6 mm) with more numerous ovules per locule (ca. 10 vs. 4-6) (Ball 1993). Due to this high similarity and presumed relationship, both taxa are considered conspecific by some authors (e.g. Dorofeev (2002)).

#### Notes

Seed set of *Armoraciarusticana* is typically poor and, for this reason, vegetative propagation strongly prevails in cultivation and secondary dispersal of the species (Sampliner and Miller 2009). This feature, along with the lack of the native distribution area, indicates that the species origin is cultigenic; phylogenetically, it may have been derived directly from *A.macrocarpa* (Miller et al. 2010).

The spread of the species by humans was connected with its medical and, later, culinary use as a spice and a digestive or condiment (Wedelsbäck Bladh and Olsson 2011). From the ethnological and linguistic information, the cultivation most likely originated in Eastern Europe (Candolle 1908). The early medicinal cultivation in Northern Europe was concentrated in monasteries in the 12^th^ century (Wedelsbäck Bladh and Olsson 2011). The peak of the European cultivation and commercial production of the species roots was observed in the 19^th^ century, after which the popularity has significantly declined (Wedelsbäck Bladh et al. 2014).

#### Introduction to Kyrgyzstan

##### Period of introduction

Neophyte.

Our record of *Armoraciarusticana* in the Sary-Chelek Nature Reserve is dated 2005. Following the report of Deza (1989), we assume that the first alien occurrences in the country were observed no later than in the 1970s-1980s, whereas its original introduction was made by the first Russian colonists.

##### Pathways of introduction

Escape from confinement: Agriculture.

The species is known exclusively as escaping from cultivation. Its further spread has not been observed.

##### Source of introduction

Eastern Europe.

The species was introduced by settlers and suppliers from Eastern Europe.

##### Invasion status

Casual.

Established populations have not been observed. The colony in the Sary-Chelek Nature Reserve is locally persisting.

##### Evidence of impact

Agriculture - no impact (not occurring as a weed). Native ecosystems - minor impact (occurrence near populated places). Urban areas - minor impact (ruderal occurrence).

##### Trend

Declining (inferred).

The popularity of *Armoraciarusticana* was noticeably fading during the second half of the 20^th^ century and now it has gone out of fashion as a garden vegetable. Nevertheless, the species keeps its ruderal presence in the territory due to its extraordinary ability for persistence and further spread with disturbed ground.

### Crambe orientalis

L., 1753

A82839A9-E1CF-5608-A8B5-CCC46A1D36B3

urn:lsid:ipni.org:names:281660-1

***Crambeorientalis*** L., Sp. Pl. 2: 671 (1753).
= *Crambeamabilis* Butkov & Majlun, Bot. Mater. Gerb. Inst. Bot. Akad. Nauk Uzbeksk. S.S.R. 17: 3 (1962).
— *Crambeschugnana* auct. non Korsh.: Vasilieva (1961).

#### Distribution

##### Native distribution

Asia Minor, Caucasus, Near East, Iraq, Iran, Turkmenistan (mountains), Afghanistan, Pakistan (Khalilov 1993).

##### Secondary distribution

Central Asia (Kazakhstan, Kyrgyzstan, Tajikistan, Uzbekistan).

##### Distribution in Central Asia

**Figure 4.**
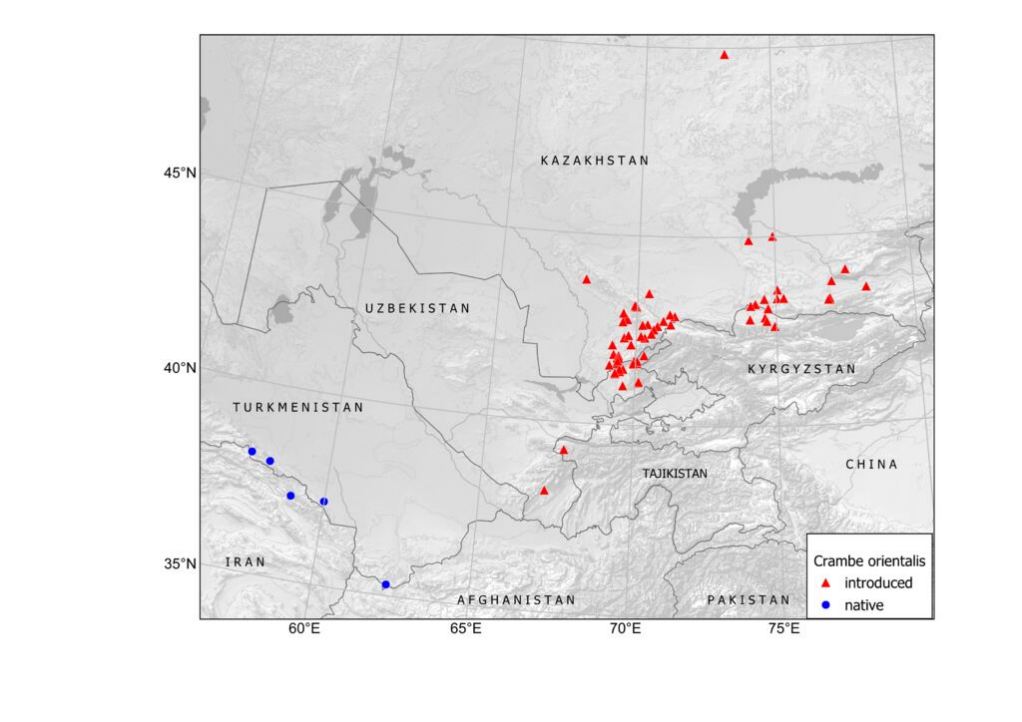
The current distribution of *Crambeorientalis* in Central Asia, according to the specimens examined, documented observations (iNaturalist 2024, Plantarium 2024) and reliable publications (Nikitin and Geldykhanov 1988, Nobis et al. 2016).

Kazakhstan, Kyrgyzstan, Tajikistan, Uzbekistan (Fig. 4).

In Kazakhstan, the species is known from a vast area along the north-western Tian-Shan (Botschantzev 1977, Lazkov and Redina 2007). It is currently known also from several localities in southern Kazakhstan from Almaty to Lake Balqaş (Nobis et al. 2016, iNaturalist 2024, Plantarium 2024) and in one isolated locality in northern Kazakhstan, Karagandy Town (Plantarium 2024).

In Uzbekistan, the species was found in the eastern part of the country, mostly adjacent to Kazakhstan (Botschantzev 1977) and recently also in an isolated locality in the south, near Boysun Town (previously unpublished record).

In Kyrgyzstan, the species was first reported from the Chüy Depression, north of Bishkek (Lazkov and Redina 2007) and then from the Talas Depression, the western part of the country at the border with Kazakhstan (Lazkov and Sennikov 2014).

In Tajikistan, the species was recently found as naturalised in agricultural areas along the Mogendarya River (Nobis et al. 2016). This locality is not connected with the native distribution area in the adjacent Afghanistan because the species has not been found along the Amudarya River in the northern part of that country (Breckle et al. 2013).

In Turkmenistan, the species is native along the mountains in the southern parts of the country (Nikitin and Geldykhanov 1988). No alien localities are known.

In Central Asia, *Crambeorientalis* was found for the first time on a fallow field at Jeri post station (Leninskoe Village in the Soviet times, Kazygurt Village in Kazakhstan) along the road from Tashkent to Şymkent, in present-day Kazakhstan, in 1922. To develop on that field, this slow-growing perennial species must have been introduced a few years before its sampling, i.e. already in the Imperial times. We do not have any written or even indirect evidence regarding the origin of this record, but it can be inferred from published side evidence. As the species is not a segetal weed, it must have been introduced for cultivation, as an edible or forage plant. Already in the mid-19^th^ century, the Caucasian Society for Agriculture (established for public edication in Tiflis, now Tbilisi, in 1850) has widely advertised the culinary values of the cultivated *C.maritima* L. and its wild relatives (Kolodeev 1853) in the Caucasus. The society offered seed material from Tiflis, which was easy to collect also in native populations of *C.orientalis*, occurring in the vicinity of the city (Grossheim 1950). The seed distributed by the society was the most likely source for the early experimental cultivation of *C.orientalis* in Central Asia.

**Figure 5.**
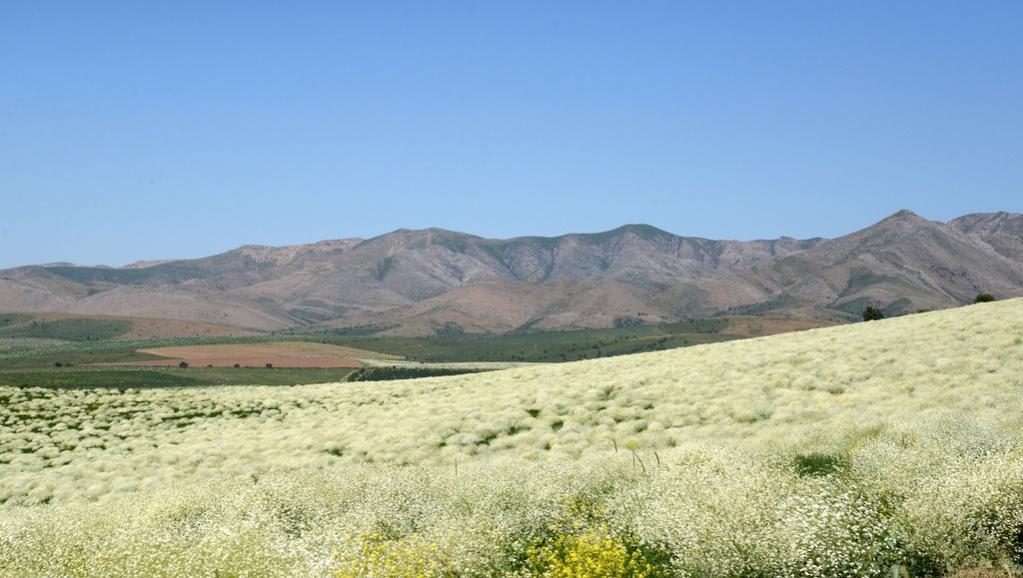
Alfalfa field with a naturalised population of *Crambeorientalis* in full blossom along the Kökbūlaq River near Pisteli Village, Türkıstan Region, Kazakhstan (photo by E. Belousov, 21 May 2021). Source: https://www.plantarium.ru/page/image/id/695395.html (Plantarium 2024).

As evident from numerous herbarium specimens collected in that area, the field at Jeri became the source of the species invasion, which also covered the neighbouring villages along the main road (Botschantzev 1977) and expanded to natural habitats (Butkov and Mailun 1962). In the 1950s, the species was found also south of Tashkent, spreading along the Chirchik and Pskem Rivers, which are adjacent to the place of the original introduction. Currently, it is widely distributed along the north-western Tian-Shan, forming extensive populations especially in arable lands (Fig. 5), but also ascending to the mountain slopes from river valleys (German, pers. obs.).

In the 1950s-1970s, *Crambeorientalis* was cultivated on experimental fields and in gardens in Uzbekistan and Kyrgyzstan as a forage or ornamental plant (Amirkhanov et al. 1974, Kadyrkulova 1987). It was recommended for cultivation and, according to herbarium records, used widely in Central Asia. Naturalised remnants of its former field cultivation were found in Uzbekistan (Boysun), Tajikistan (Mogendarya) and Kyrgyzstan (Chüy Depression), whereas its former ornamental cultivation, documented in Uzbekistan (Tashkent, Yangidarya), Kyrgyzstan (near Bishkek) and Kazakhstan (Almaty, Karagandy), also produced locally established populations.

Although the field and garden use of the species is no longer popular, it continues spreading from the places of its former cultivation. Its expansion, well documented in many areas, shows a high potential for the future invasion.

##### Distribution in Kyrgyzstan

**Figure 6.**
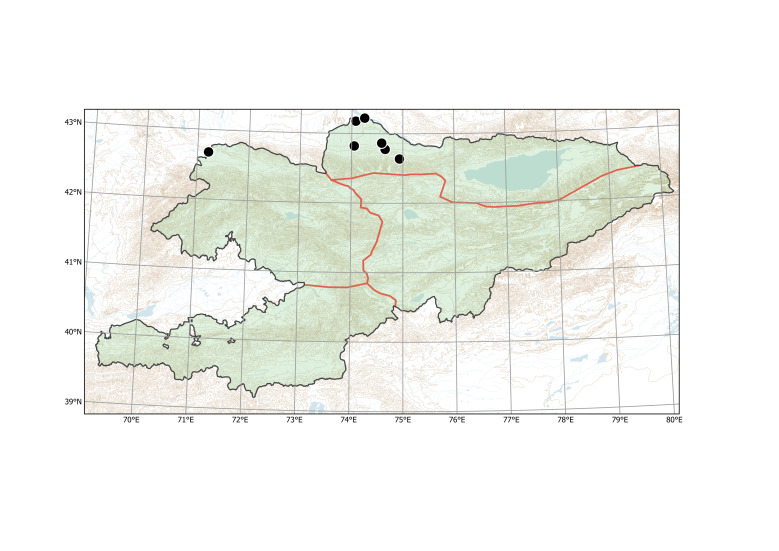
The current distribution of *Crambeorientalis* in Kyrgyzstan.

Western Tian-Shan, Northern Tian-Shan (Fig. 6).

**Figure 7.**
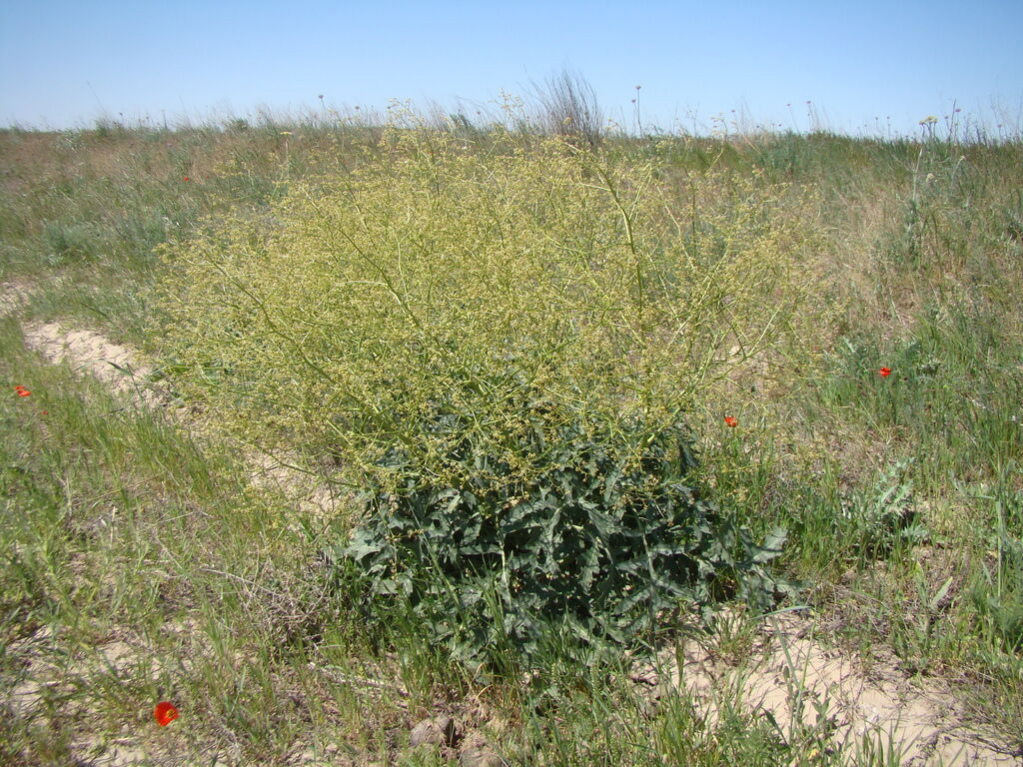
Ruderal occurrence of *Crambeorientalis* along an irrigation ditch in the field area near Kamyshanovka Village, Chüy Region, Kyrgyzstan (photo by G. Lazkov, 11 July 2011).

The species is known from two separate areas in the Western and Northern Tian-Shan. The single locality in the Western Tian-Shan, discovered in 2013 (Lazkov and Sennikov 2014), is a direct continuation of the first invasion that started between Tashkent and Şymkent. Several localities around and north of Bishkek belong to an area of the species invasion in the Chüy Depression (Fig. 7), which has been repeatedly sampled since 2006 (Lazkov and Redina 2007).

The species was found mostly on the plain and in foothills, but also cultivated in gardens at elevations up to 1600 m a.s.l.

#### Ecology

Dry stony slopes on plains and in foothills in the native distribution area. Ruderal places, fallow and cultivated fields in the secondary distribution area.

#### Biology

Perennial with a strong, thickened taproot, forming a tumbleweed structure that facilitates self-dispersal for considerable distances.

In cultivation, the plants start flowering in the third year. Native occurrence and cultivation do not require any special water supply, so that the plants may develop without irrigation in arid territories.

#### Taxon discussion

*Crambeorientalis* was not known to Botschantzev and Vvedensky (1955) due to the lack of earlier herbarium collections from Uzbekistan in Tashkent. The species was rediscovered in the field along the Keles River and between Angren and Chirchik rivers by Butkov and Mailun (1962), who did not recognise its correct identity and ventured to describe it as a narrow endemic to Uzbekistan, *C.amabilis*. Botschantzev (1977) observed the species in its type locality and realised its alien origin; he identified the species as *C.orientalis* and this identity has been widely accepted (Kovalevskaya 1974, Khalilov 1993, POWO 2024).

#### Notes

Czernjakowska (1939) reported the traditional use of *Crambeorientalis* as an edible plant, but that record was erroneously based on the old report of Hablitz (1785), who described the use of the Crimean plants previously referred to this species. However, the active use of another, ecologically similar species in Central Asia has been recorded: *C.kotschyana* Boiss. was traditionally used for fodder in Uzbekistan (Amirkhanov and Solonov 1964) and Afghanistan (Vavilov and Bukinich 1929) and as edible and fodder in Turkmenistan (Larin and Larina 1951). In the foothills of the North Caucasus, the culinary use of "*C.tatarica*" (= *C.grandiflora* DC.) as a substitute for horseraddish was recorded in Georgievsk (Kolodeev 1853).

*Crambeorientalis* was evaluated and subsequently tried as a commercial fodder plant in Central Asia during the 1950s-1980s (Amirkhanov et al. 1974, Kadyrkulova 1987). Numerous authors stressed the aesthetic value of the plant, for which it was widely cultivated in private gardens in Central Asia during the same period.

#### Introduction to Kyrgyzstan

##### Period of introduction

Neophyte.

The species was originally introduced In the late Soviet period, when the plants were used in experimental field cultivation (Kadyrkulova 1987). In the 2000s, naturalised populations have already been found.

##### Pathways of introduction

Escape from confinement: Agriculture. Escape from confinement: Ornamental purpose other than horticulture. Unaided: Natural dispersal across borders.

The primary way of the species introduction was its cultivation on fields for fodder and in private gardens as ornamental plants. The species is known to run wild from the places of original cultivation and this was the cause of its introduction in the Chüy Depression (from fields) and around Bishkek (from gardens).

The secondary dispersal of the species occurs actively by wind. This was the cause of introduction in the Talas Depression in Kyrgyzstan, to which the plants have arrived from the neighbouring areas in Kazakhstan.

##### Source of introduction

Caucasus.

Native Caucasian populations were accessible as a source of cultivation in the Russian Empire and the USSR.

##### Invasion status

Naturalised.

The species is apparently naturalised in the Chüy Depression, where it has been repeatedly observed for nearly 20 years.

##### Evidence of impact

Agriculture - minor impact (may occur in arable lands). Native ecosystems - minor impact (may colonise natural landscapes from agricultural lands). Urban areas - minor impact (sometimes found along roadsides and in ruderal places).

##### Trend

Increasing (observed and inferred).

The species actively expands in Central Asia. Its recorded occurrence in Kyrgyzstan has also been growing constantly.

### Hesperis pycnotricha

Borbás & Degen, 1902

1EBA1E65-3E8B-558C-BDBE-5F968371CDE4

urn:lsid:ipni.org:names:20011225-1

***Hesperispycnotricha*** Borbás & Degen, Magyar Bot. Lapok 1: 269 (1902).
— *Hesperismatronalis* auct. non L.: Deza (1989), Lazkov and Sultanova (2011), Lazkov and Sultanova (2014).
It is commonly believed (IPNI 2024) that the species name *Hesperispycnotricha* was validly publised in 1903, when the species was described in full and in Latin language (Borbás 1903, p. 17). Its correct nomenclatural citation was provided by Kotov (1979), who noted that the conditions for valid publication (species diagnosis in Hungarian language, in an identification key) were fulfilled earlier, in a preceding part of the same article (Borbás 1902, p. 269).

#### Distribution

##### Native distribution

The species distribution covers south-eastern Europe (Bulgaria), southern part of Eastern Europe (including the neighbouring parts of Slovakia), north-western Caucasus and Asia Minor (Tzvelev 1959, Kotov 1979, Jalas and Suominen 1994).

##### Secondary distribution

Europe (Tzvelev 1959, Jalas and Suominen 1994), Northern Asia (Chepinoga et al. 2024), North America (Dorofeev 2013).

The species has been extensively cultivated in Eastern Europe (Tzvelev 1959, Kotov 1979), Siberia (Tzvelev 1959) and known as escaping from cultivation in these territories (Tzvelev 1959, Kotov 1979). Its occurrence in Central Asia remained very obscure until Lazkov et al. (2011) reported an alien record from Kyrgyzstan. Our data suggest that this is the only species of the *H.matronalis* group that is commonly cultivated in Central Asia.

According to the herbarium specimens cited by Tzvelev (1959), the species has been cultivated in Eastern Europe at least since the mid-19^th^ century and found as escaped from cultivation in Siberia (Omsk Town) in 1886. Although it was common in ornamental cultivation already in the 19^th^ century, its alien occurrence in Siberia has been registered rather recently; to date, in Northern Asia, it is known from Western Siberia and Altai (Chepinoga et al. 2024).

In North America, the species is most common among the cultivated and alien members of the *H.matronalis* group, which is widely naturalised in the USA (Rollins 1981) and occurs in many states of the USA and also in Canada (Dorofeev 2013).

##### Distribution in Central Asia

**Figure 8.**
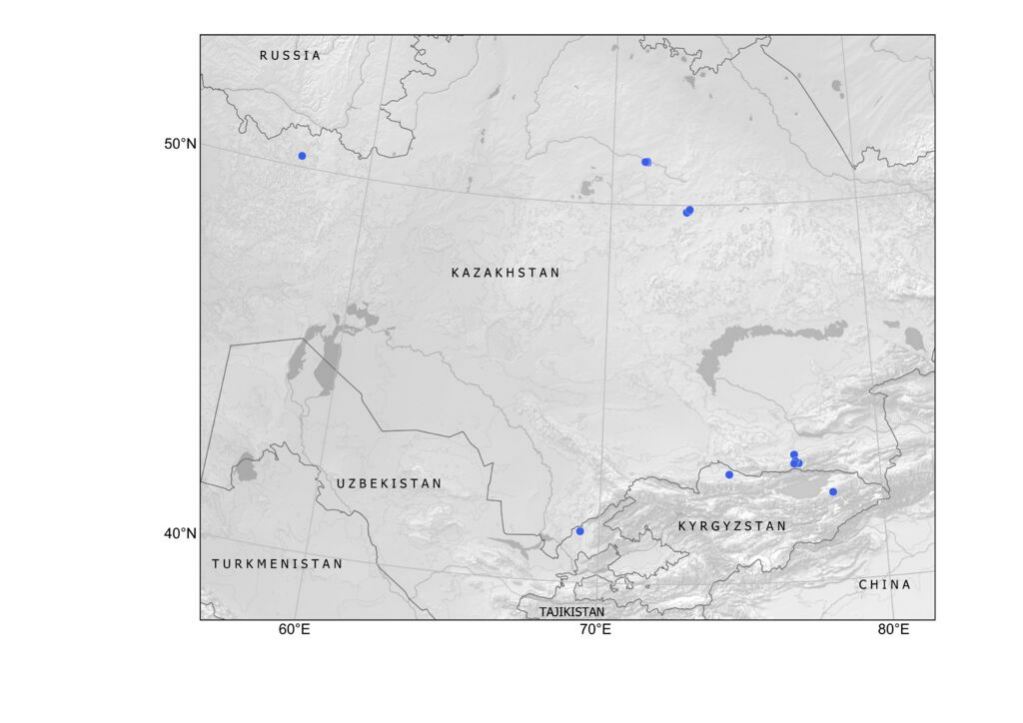
Cultivation and subspontaneous occurrence of *Hesperispycnotricha* in Central Asia according to the specimens examined and documented observations (iNaturalist 2024, Plantarium 2024).

Kazakhstan, Kyrgyzstan, Uzbekistan (Fig. 8).

**Figure 9.**
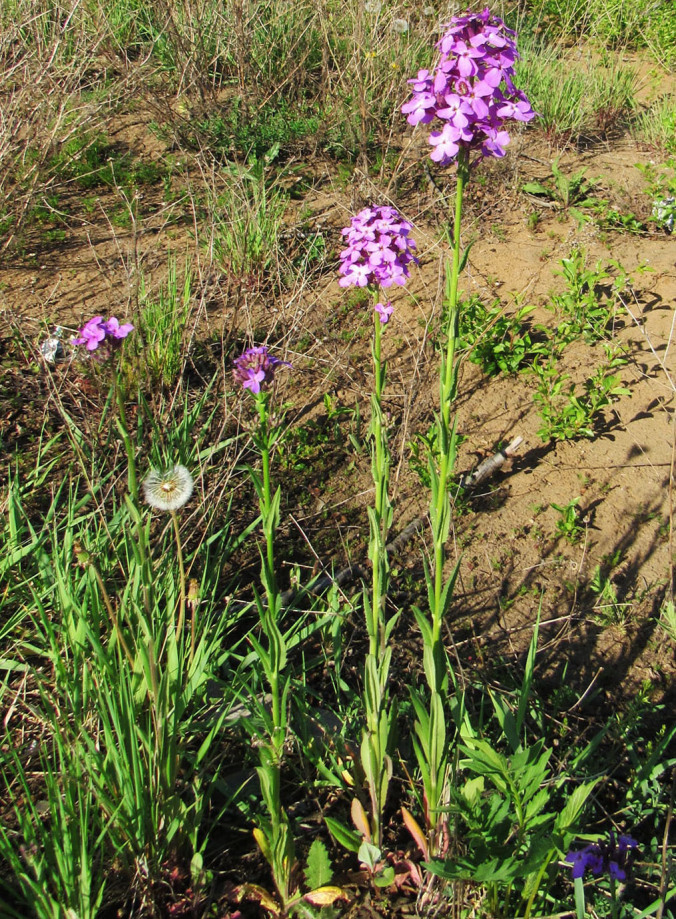
A ruderal occurrence of *Hesperispycnotricha* (pink flower variant) in Karagandy Town, Kazakhstan (photo by I. Evdokimov, 13 May 2020). Source: https://www.plantarium.ru/page/image/id/656303.html, misidentified as *H.matronalis* (Plantarium 2024).

As evident from herbarium collections, the species was cultivated in southern Kazakhstan since the last decades of the 19^th^ century ("Flora iliensis" = Ili River Basin [most likely Almaty Town], 1886, A.N.Krasnov (LE)), i.e. from the beginning of its settlement by Russian colonists. The long tradition of ornamental cultivation suggests that the species is present in ruderal habitats. Such occurrences (Fig. 9) have been found on online citizen-science platforms (iNaturalist 2024, Plantarium 2024) and provide evidence for the species naturalisation or long-time persistence in the country. This is the first record of the subspontaneous occurrence of *Hesperispycnotricha* in Kazakhstan.

In Uzbekistan, the species is cultivated in populated places, but not considered as running wild (Sennikov et al. 2020). Its first herbarium collection from cultivation is dated by the 1930s (Tashkent, in a garden of P.A.Baranov, 19.06.1932, A.Lapin (LE)).

In Kyrgyzstan, *Hesperispycnotricha* was reported as being cultivated and occasionally escaped from cultivation under the wrong name *H.matronalis* (Deza 1989). Its later record as new to Kyrgyzstan and Central Asia as a whole (Lazkov et al. 2011) was the first documented observation of the species dispersal. The occurrence of the species in a spruce forest of the Jety-Ögüz River ravine (Lazkov et al. 2011) may look strange when considered isolated from the context because it misleadingly hints at the wilderness. The ravine is a popular touristic attraction and a path along the river starts from the sanatorium that is famous for its geothermal springs. The species was apparently dispersed to the wild from flowerbeds in touristic places.

In Tajikistan, the species is apparently cultivated, but we have no documentation of its occurrence.

##### Distribution in Kyrgyzstan

**Figure 10.**
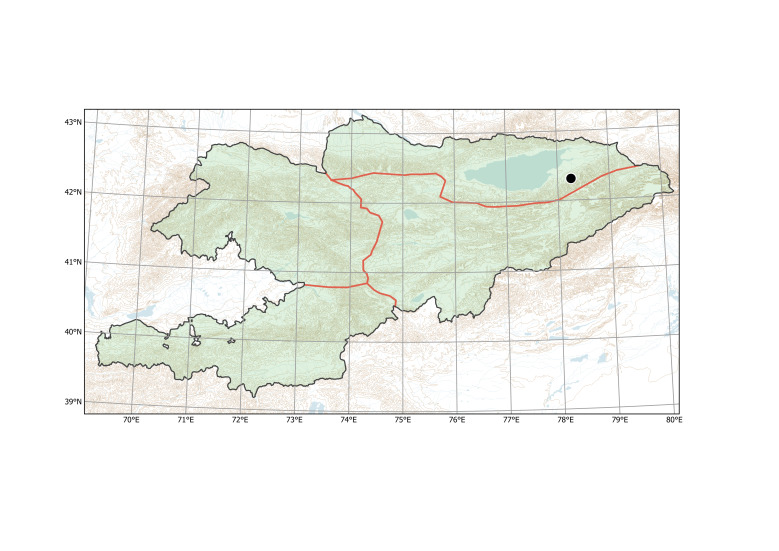
The subspontaneous record of *Hesperispycnotricha* in Kyrgyzstan according to the specimen examined.

Northern Tian-Shan (Fig. 10).

#### Ecology

Meadow steppes and true steppes in lowlands and foothills in the native distribution area (Tzvelev 1959). Ruderal places, roadsides, riversides in the secondary distribution area.

#### Biology

Biennial plant with a small taproot.

#### Taxon discussion

The taxonomy of the *Hesperismatronalis* group largely relies on flower colour and pubescence; species rank is commonly accepted for the main segregate taxa. The following three species are involved in a taxonomic confusion in Central Asia (Tzvelev 1959, Kotov 1979, Ball 1993).

*Hesperismatronalis* L. s.str. is a mesophytic species of forest meadows, which is characterised by the pubescence of simple hairs. Its upper leaves are gradually attenuated into a very short petiole. Native distribution: Europe, Caucasus, Asia Minor.

*Hesperispycnotricha* is a xerophyte occurring mostly in steppes, which has a dominant pubescence of short branched hairs, sometimes with scattered short simple hairs. Its upper leaves are sessile, slightly amplexicaul. Native distribution: centred around the Black Sea.

*Hesperissibirica* L. is a mesophyte largely associated with coniferous forests, with dominant simple hairs and a glandular pubescence in the inflorescence (sometimes covering the whole plant). Its upper leaves are sessile, sometimes auriculate. Native distribution: Central Asia, Northern Asia (including parts of Mongolia and China).

The pubescence type and the shape of leaves clearly discriminate the three taxa and their different native distribution areas and ecological preferences confirm the distinction. *Hesperismatronalis* and *H.pycnotricha* were commonly treated as a single species in the past, but that confusion has survived until recent times in non-taxonomic literature (e.g. Deza (1989)) and some taxonomic databases (e.g. POWO (2024)).

The taxonomic distinction among these three taxa allows us to separate native (*H.sibirica*) and non-native (*H.pycnotricha*) occurrences of the *H.matronalis* group in Central Asia. Surprisingly, *H.matronalis* s.str. is completely absent from the Central Asian herbarium collections and documented observations. Its previous reports from the local cultivation (e.g. Deza (1989), iNaturalist (2024), Plantarium (2024)) are based on the broad species treatment and should be interpreted as belonging to *H.matronalis* s.l. According to our examination, the actual material identified as *H.matronalis* belongs exclusively to *H.pycnotricha*. For this reason, *H.matronalis* s.str. should be excluded from the flora of Central Asia.

#### Notes

The flower colour varies noticeably within the species. The most common variety has pink flowers, whereas a darker, pinkish-violet flower variant can also be found (Tzvelev 1959, Kotov 1979, iNaturalist 2024).

#### Introduction to Kyrgyzstan

##### Period of introduction

Neophyte.

The species was introduced most likely during the same period as in Uzbekistan and Kazakhstan, i.e. the last quarter of the 19^th^ century, due to the common market of garden cultivation in the Russian Empire. The beginning of its running wild is uncertain, but ruderal occurrence is highly likely from the beginning of cultivation.

The first published observation of its alien status belongs to the 1980s (Deza 1989) and the first observation in native habitats is dated 2009 (Lazkov et al. 2011).

##### Pathways of introduction

Escape from confinement: Ornamental purpose other than horticulture.

The plants were cultivated outdoors for ornamental purposes and run wild from the places of cultivation.

Seed dispersal on human feet is a likely vector of the secondary dispersal, as the species is often observed growing along pedestrian paths.

##### Source of introduction

Eastern Europe.

The species has been repeatedly introduced via the ornamental seed supply of the Russian Empire and the USSR.

##### Invasion status

Casual.

The ruderal occurrences of the species in Kyrgyzstan (Deza 1989) should be casual as no such naturalisation is currently known. The species status in a single locality recorded in the Teskey Alatoo (Lazkov et al. 2011) is unknown as the locality description is unavailable. We prefer to treat the species status as casual (Sennikov and Lazkov 2024a) until naturalised populations or at least persisting colonies are known.

##### Evidence of impact

Agriculture - no impact (not recorded in crop production areas). Native ecosystems - minor impact (once recorded in recreation forest areas, may be found elsewhere outside populated places). Urban areas - minor impact (sometimes escapes and occurs in ruderal places).

##### Trend

Slowly increasing (inferred).

The species has long been highly popular in ornamental cultivation. Its wide use for flower beds and in private gardens constantly increases the risk of unintentional introduction. Further discoveries of locally persisting or even naturalised populations are expected, as evident from the current expansion of the species in Siberia (Ebel 2002).

### Hirschfeldia incana

(L.) Lagr.-Foss., 1847

DD1846F9-7EA2-5A99-80E9-936A122EE013

urn:lsid:ipni.org:names:285350-1

***Hirschfeldiaincana*** (L.) Lagr.-Foss., Fl. Tarn Garonne: 19 (1847) — *Sinapisincana* L., Cent. Pl. I: 19 (1755).

#### Distribution

##### Native distribution

Mediterranean, including north-western Africa, south-western Europe, Italy, Greece, Crimea, Asia Minor, Near East, Transcaucasia, Iran and south-western Turkmenistan.

##### Secondary distribution

Europe, South Africa, temperate North and South America, Australia, scattered localities in Asia.

In North America, the species was noted in California first in 1895 and as a noxious weed in the 1930s (Jepson 1936). It is widely naturalised in the western states of the USA (Rollins 1981) and Mexico (Villaseñor and Espinosa‐Garcia 2004).

In South America, the species has developed an extensive occurrence in argicultural areas (GBIF 2024), which is also connected with the species dispersal with imported wheat to Europe, as recorded in Finland already in the 1950s (Suominen 1979).

In Australia, *Hirschfeldiaincana* was introduced in the late 19^th^ century and became naturalised in the very beginning of the 20^th^ century. Its significant field occurrence and grain contamination has been observed in the south-eastern provinces (Parsons and Cuthbertson 2001), causing the species immigration to Finland with Australian contaminated wheat in the 1950s (Suominen 1979).

The territories of the extensive naturalisation of *Hirschfeldiaincana* correspond to grain production areas, thus indicating its major distribution pathway with imported grain. Grain import as a major pathway is indicated by the species occurrence along railway tracks, for example, in Lithuania (Gudžinskas 1997) and North-Western European Russia (Tzvelev 2000). The secondary dispersal takes place along roadsides by attaching seeds to vehicles and feet, by road construction and with flowing water (Parsons and Cuthbertson 2001).

A recent expansion of the species in Britain is connected with the increasing trade of contaminated bird seed since the 1990s (Patel 2004). In addition to the previous spread of the species by bread grain trade, we suggest that its recent global dispersal may be largely caused by the increasing production of poultry in the Developing World since the 1990s (Narrod et al. 2008), which requires extensive import of grain food that is not sufficiently available in arid countries of Asia, whose climate may be suitable for the species naturalisation.

The exact extension of the secondary spread of *Hirschfeldiaincana* may not be properly documented because its occurrences are largely ephemerous due to its high demand for warm temperatures; this can be demonstrated with an example of Finland in which numerous species records exist because of the dedicated sampling for exotic alien plants (Suominen 1979). Outside the areas with high summer temperatures, its populations are mostly temporarily persisting and not really established.

##### Distribution in Central Asia

**Figure 11.**
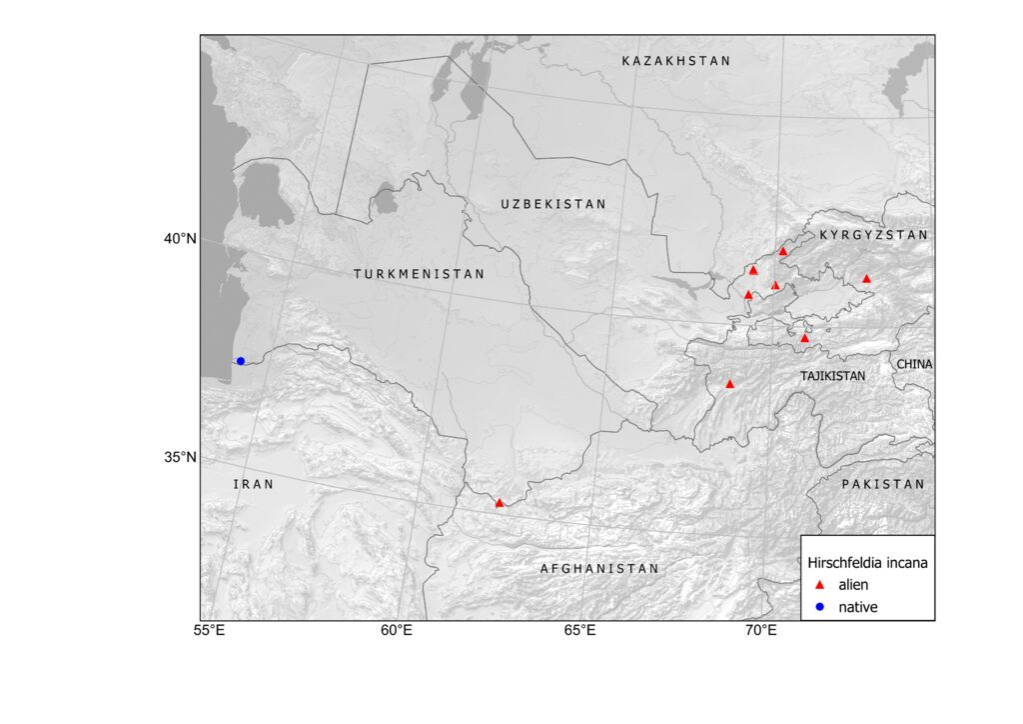
The native and alien records of *Hirschfeldiaincana* in Central Asia, according to herbarium specimens examined and reliable literature (Nardina 1954).

Kyrgyzstan, Tajikistan, Turkmenistan, Uzbekistan (Fig. 11).

In Central Asia, the species was first found in Turkmenistan in 1946, in a single locality at Ajyýap Village, present-day Esenguly District, Balkan Province, along field margins and in saline lands (Nardina 1954), although the voucher specimen is currently lacking (Voitenko 1969). In this area, segetal weeds were numerous and especially abundant along the river (Nardina 1954), thus showing a developed agricultural activity.

**Figure 12.**
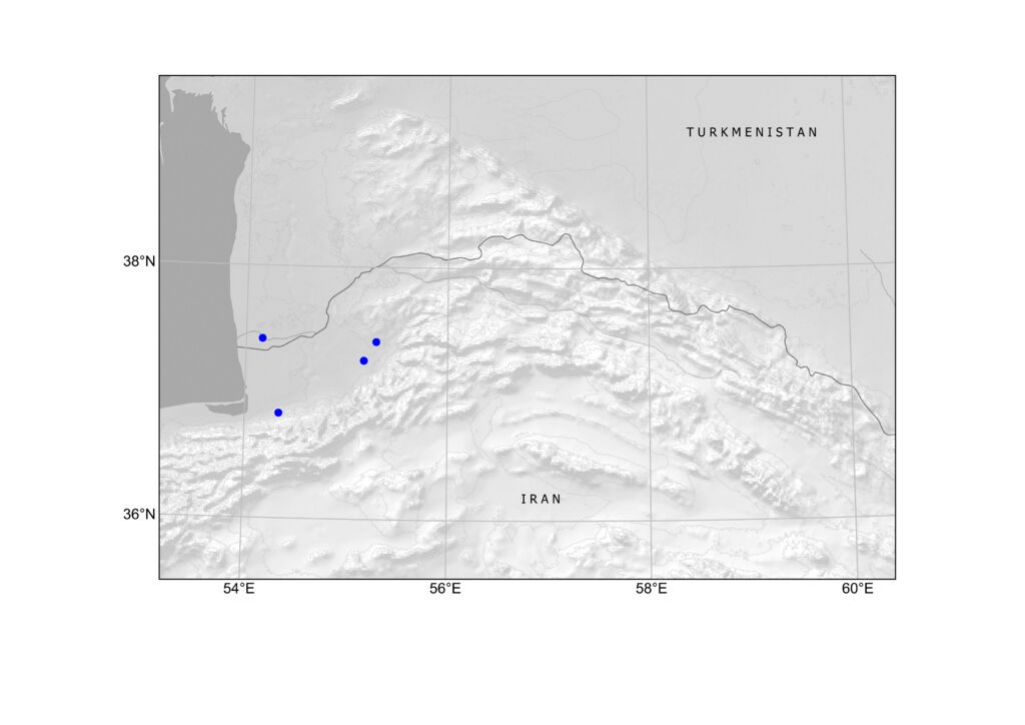
The presumably native distribution of *Hirschfeldiaincana* in north-eastern Iran and Turkmenistan, according to Nardina (1954) and Hedge (1968).

The first record of *Hirschfeldiaincana* in Turkmenistan seems to have been connected with an agricultural region in Turkmen Sahra, Golestan, Iran, a territory historically inhabited by the Turkmen people who were otherwise largely nomadic. Herbarium records from this region uncovered a continuous distribution of the species (Fig. 12). This distribution strongly suggests that the species was present in the territory before the Russian conquest of Turkmenistan. Its status can be inferred as presumably native in south-western Turkmenistan because of its numerous occurrences in the lowlands of Turkmen Sahra, not only as a weed, but also in native plant communities and natural landscapes, as noted by Nardina (1954).

One more historical locality, along the railway near Kushka Station, was observed in 1968 (Voitenko 1969). This occurrence is apparently connected with the imported wheat grain, from the times of the Soviet food crisis (Volin and Walters 1965, Moravcik 1978); otherwise, the species would have been noticed in that place earlier, in the course of its botanical exploration since the early 20^th^ century.

In Kyrgyzstan, the species was first observed as a casual alien in Kyr-Koo Village in 2013 (Lazkov and Sennikov 2014). The second record, dated 2022, is reported in the present contribution.

In Tajikistan, the species has been recorded only once, as a recent casual introduction in a ruderal place in Dushanbe City, in 2019 (Ebel et al. 2022).

The species records in Uzbekistan are most recent in Central Asia (German et al. 2024). In a single year, during 2024, it has suddently emerged in several localities along roads and on city lawns in Tashkent and north-eastern parts of Uzbekistan, often as groups of several individuals. We assume that this introduction has originated from the recent import of foreign fodder grain.

##### Distribution in Kyrgyzstan

**Figure 13.**
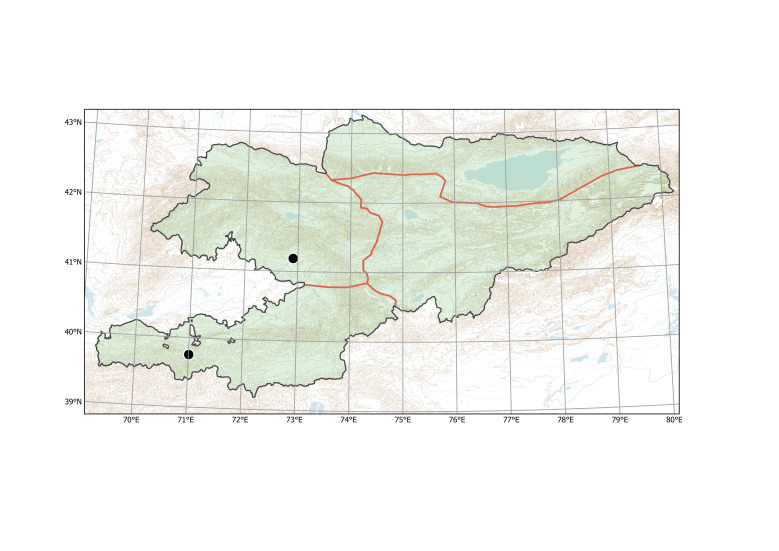
The documented occurrence of *Hirschfeldiaincana* in Kyrgyzstan.

Western Tian-Shan, Alay-Turkestan (Fig. 13).

**Figure 14.**
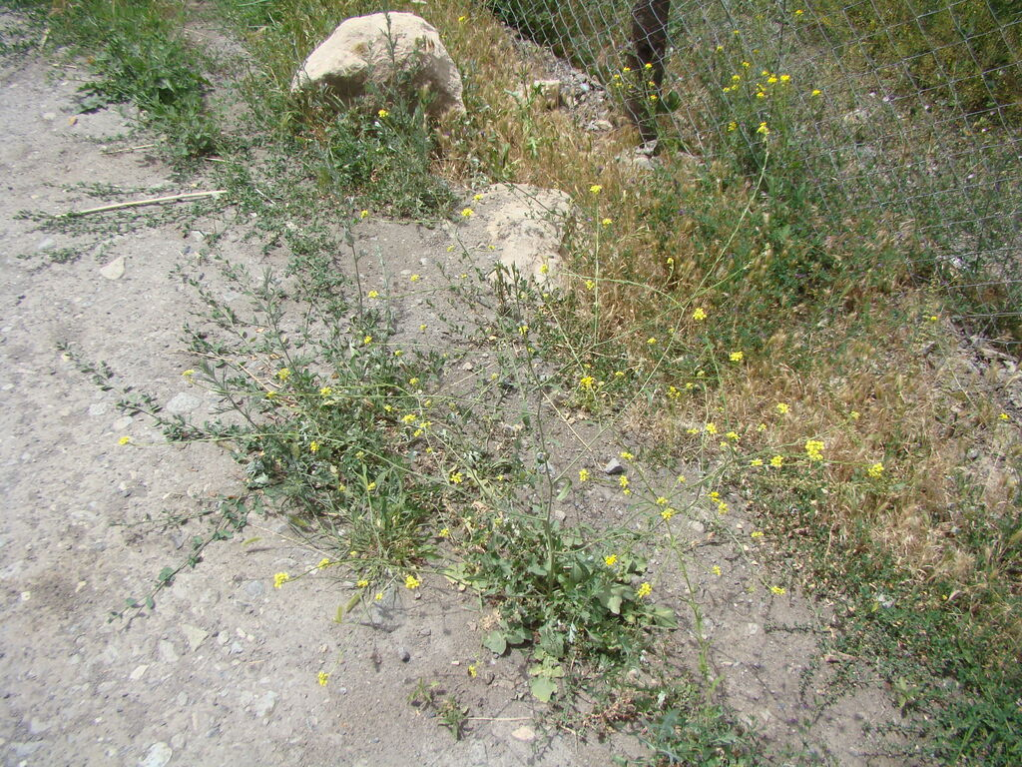
*Hirschfeldiaincana* in Sary-Talaa Village, Jalal-Abad Region, Kyrgyzstan (photo by G. Lazkov, 5 July 2022).

After the first record (Lazkov and Sennikov 2014), the species has been recently observed in Sary-Talaa Village in south-western Kyrgyzstan (Fig. 14).

#### Ecology

Open stony, gravelly, sandy and other dry places in the native distribution area; possibly, the species was originally confined to sandy and gravelly sea shores (Tzvelev 1977). Nutrient-rich soils are preferred (Hanf 1983).

#### Biology

Biennial to perennial, with a slender taproot.

In germination experiments, fruit valves were found to induce seed dormancy, which leads to a seed soil deposit originating from an indehiscent apical segment of the fruit, allowing the species to survive periods of drought and successfully colonise the territory (Voitenko 1969). A strong relation of seed dormancy with seed size has been recently observed (Mira et al. 2019).

The requirement for warm temperatures in germination (the maximal germination rate was found in experiments with temperatures between 20 and 35°C: Castro et al. (2015)) seems to limit the species naturalisation to the areas with a relatively hot climate.

#### Taxon discussion

The generic position of the species and, consequently, the generic status of *Hirschfeldia* were recently challenged (Al-Shehbaz 2018). Based on the comparative genome studies, it has been established that *Hirschfeldia* is an ancient intergeneric hybrid lineage that originated from the crosses between *Brassica* L. and *Mutarda* Moench (Hoang et al. 2024).

The genetic diversity in naturalised populations of the British Isles was found as high as in the native populations and also lacking any apparent spatial structure, thus showing multiple and repeated introduction events that presumably enable the species to colonise new territories (Lee et al. 2004).

The species may produce hybrids with *Brassicanapus* L., but without successful backcrossing (Darmency 2002).

#### Notes

**Figure 15.**
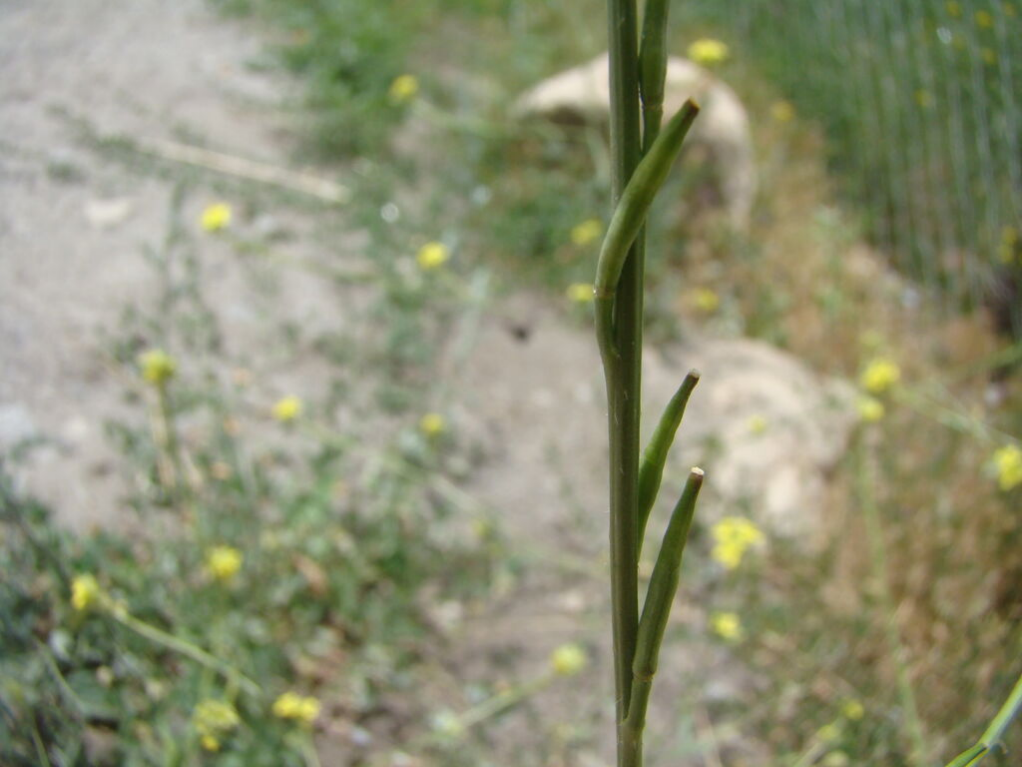
A plant of *Hirschfeldiaincana* with a short and nearly straight beak of the pods in Sary-Talaa Village, Jalal-Abad Region, Kyrgyzstan (photo by G. Lazkov, 5 July 2022).

Tzvelev (1977) examined the variability of *Hirschfeldiaincana* in the Crimea and the Caucasus and concluded that three subspecies can be distinguished in that area on the basis of fruit variability (in length, width and lignification of the pods, length and direction of the beak). Most notably, he separated H.incanasubsp.leptocarpa Tzvel. because of its long and slender pods with a slender and recurved beak; such plants can be found in the same area as the type subspecies occurring from the Near East to the Transcaucasia, without any spatial pattern and may be best treated at the level of variety, as H.incanavar.geniculata (Desf.) Bonnet & Barratte. The plants from Central Asa have shorter pods with a short and straight beak, thus belonging to the type variety (Fig. 15).

#### Introduction to Kyrgyzstan

##### Period of introduction

Neophyte.

The species has been observed during the independence time, since 2013.

##### Pathways of introduction

Transport - Contaminant: Seed contaminant.

Both localities observed in Kyrgyzstan are linked with the import of contaminated grain. The species occurrence along ruderal roadsides strongly suggests its immigration without agricultural activities.

##### Source of introduction

According to the recent reports of the National Statistic Committee of the Kyrgyz Republic, reproduced in the local news media, Kyrgyzstan largely depends on the massive import of fodder for local farming, also from remote areas like Iran. Fodder grain is used, for example, for feeding chickens in the villages and we assume this country as a likely source of introduction because *Hirschfeldiaincana* is broadly distributed in Iran (Hedge 1968).

##### Invasion status

Casual.

The ruderal roadside occurrences observed in Kyrgyzstan are limited in size and do not demonstrate that the species has been naturalised and formed sustainable populations.

##### Evidence of impact

Agriculture - no impact (not found in cultivated lands). Native ecosystems - no impact (restricted to populated places). Urban areas - minor impact (rarely occurs in ruderal places).

##### Trend

Increasing (observed and inferred).

With the latest spread of the species in Uzbekistan (German et al. 2024), we predict that further occurrences may emerge in the future because of the continuous import of contaminated grain fodder. However, the species naturalisation is not expected in arid areas of Kyrgyzstan.

### Mutarda arvensis

(L.) D.A.German, 2022

A16527D9-0BBF-5289-A0BC-3A8592FDFB76

urn:lsid:ipni.org:names:77313885-1

***Mutardaarvensis*** (L.) D.A.German, Turczaninowia 25(2): 56 (2022) — *Rhamphospermumarvense* (L.) Andrz. ex Besser, Enum. Pl.: 83 (1822) — *Sinapisarvensis* L., Sp. Pl. 2: 668 (1753).

#### Distribution

##### Native distribution

From the species ecology and details of its distribution, we infer that the native distribution area of *Mutardaarvensis* is the Mediterranean. Its eastern distribution limit is uncertain, but apparently does not include Turkmenistan, in which the species is considered exclusively alien and originally occurring as a segetal weed in irrigated places of lowlands and foothills (Nikitin and Geldykhanov 1988).

##### Secondary distribution

Europe, temperate Asia, temperate Africa, temperate North and South America, non-desert Australia. It is found northwards as far as Iceland and the Faroes in the Arctic (Fogg 1950, Wasowicz et al. 2019). Common in North America (Rollins 1981).

The species is a common weed of many crops (Yarmolenko and Vasilczenko 1934) since the early periods of agriculture (Fogg 1950) and was recorded on traditional fields of bread crops and flax in Afghanistan (Vavilov and Bukinich 1929). Its high level of seed production and a good adaptation to arable lands brings its distribution to the limits of argicultural zone; its highest abundace is recorded in lowlands and on chernozem soils (Yarmolenko and Vasilczenko 1934), but the occurrence extends to the higher elevations with the cultivation of cereals (Fogg 1950). Its typical pathway of introduction is infestation of crop seeds and argicultural commodities (Yarmolenko and Vasilczenko 1934, Suominen 1979), making it commonly occurring also in waste and ruderal lands and along roadsides in populated places. This pathway is still active, although apparently decreasing because of the modern purification of grain crops (Vainorienė and Gudžinskas 2009). Due to its enormous seed deposit, the species commonly occurs on fallow fields, but disappears when the fields are turned into pastures (Chippindale and Milton 1934).

##### Distribution in Central Asia

**Figure 16.**
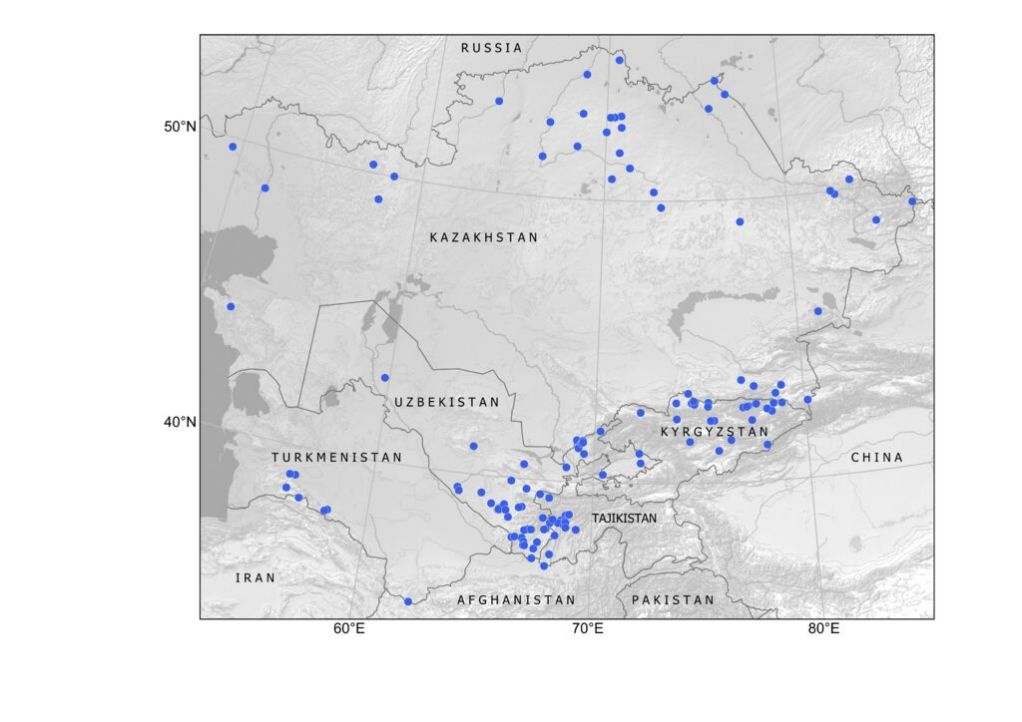
Recorded distribution of *Mutardaarvensis* in Central Asia, according to herbarium specimens, documented observations and literature data (Yunusov 1978).

The species occurs as a common alien plant in all the countries of Central Asia (Yarmolenko and Vasilczenko 1934, Kovalevskaya 1974). It occurs largely in agricultural areas, in the steppe zone in Kazakhstan and on irrigated lands in the mountains, largely avoiding deserts (Fig. 16) due to its rather high demand for water supply (Yarmolenko and Vasilczenko 1934).

The spread of *Mutardaarvensis* to Central Asia has occurred in several major waves from different directions.

Fedtschenko (1902), when visited Samarqand immediately after the Russian conquest, collected the species around the city in 1869. In Uzbekistan, as well as elsewhere in the mountainous Central Asia, the species is an archaeophyte that had arrived with the introduction of agriculture in the Neolithic period and its population in the southern Central Asia was apparently self-sustainable in agricultural lands for a long time. During the Imperial and Soviet times, the development of extensive agricultural regions, which were largely specialised on cotton and grain production, resulted in the abundant occurrence of *Mutardaarvensis* in southern Uzbekistan, south-western Tajikistan, Fergana Depression and northern Kyrgyzstan (Fig. 16).

Northern Kazakhstan, embracing the steppe areas of Central Asia, was a nomadic region, into which the species has arrived with the agriculture introduced by Russian settlers. Slovtsov (1897), who travelled across Akmolinsk steppes (present-day Kökşetau District, Astana Region, Kazakhstan) in 1878, observed that permanent settlements with agricultural activities were few and belonged to Russian Cossacks who served as frontier guards along the Siberian frontier and in its foreposts since the second half of the 18^th^ century; two early herbarium collections of the species from Kazakhstan (dated 1834 and 1878) originated from the Cossack settlements. In 20 years after this observation, northern Kazakhstan has been settled by many tens of thousands of Russian peasants, who established numerous villages and extended the cultivated lands, thus producing the second wave of the species invasion (Katanaev 1897). The third wave originated from the machinery-supported expansion of agriculture into the Asian part of the USSR (Katkoff 1950, Durgin 1962), which appeared in periods of the intensive ploughing up of previously uncultivated lands in Siberia and northern Kazakhstan in 1928-1940 and culminated with the Virgin Lands campaign of 1954-1963.

According to herbarium collections, in Central Asia, the species was found in connection with all kind of fields: wheat, barley, oat, flax, alfalfa, cotton, vegetable, beetroot. It was also found on city lawns and among ornamental plants.

The ruderal occurrence of *Mutardaarvensis* in contemporary populated places of Central Asia is complementary to the other factors. Its origin comes partly from grain contamination, partly from the infestation of ornamental cultivation or greenery.

The major floristic sources reported that the species is commonly found in Central Asia as a field weed, on fallow fields, as a ruderal plant in populated places and along roadsides (Botschantzev and Vvedensky 1955, Nikitina 1955, Vasilieva 1961, Kovalevskaya 1974, Yunusov 1978). This information reflects its major role as a segetal weed and does not provide for its naturalisation in native habitats. According to more recent observations, *Mutardaarvensis* has been repeatedly found as dispersed outside fields and ruderal places to the neighbouring riversides and ravines. These observations show the species capacity to establish in natural habitats, although to a limited extent.

##### Distribution in Kyrgyzstan

**Figure 17.**
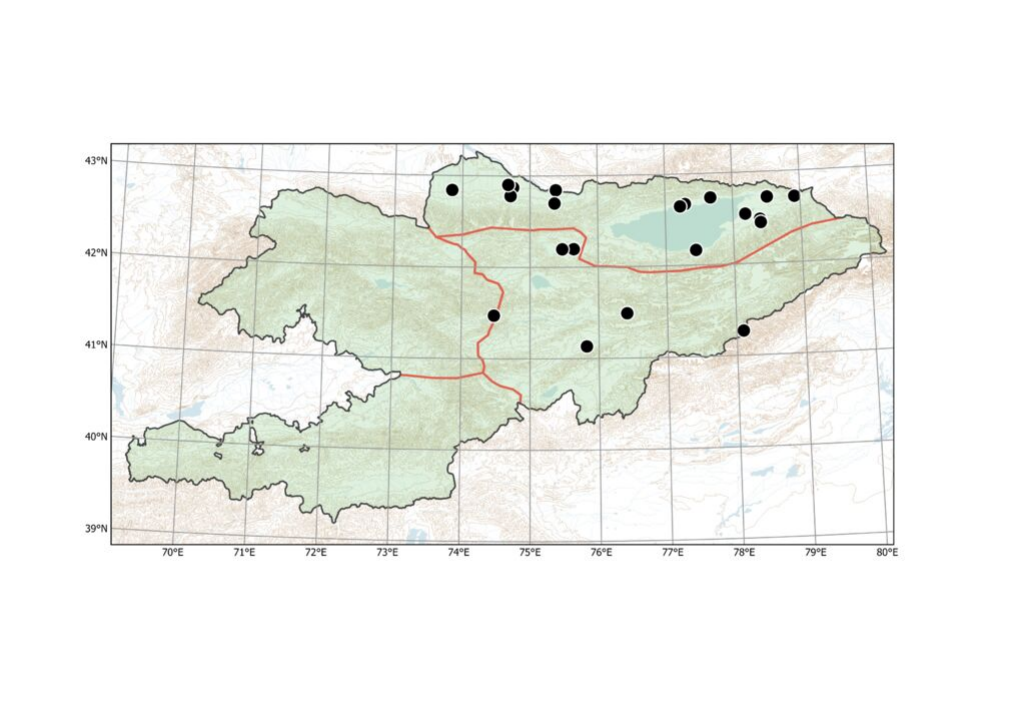
Documented records of *Mutardaarvensis* in Kyrgyzstan.

The species was found in all agricultural regions of the country (Nikitina 1955), but its current documentation is apparently deficient (Fig. 17).

**Figure 18.**
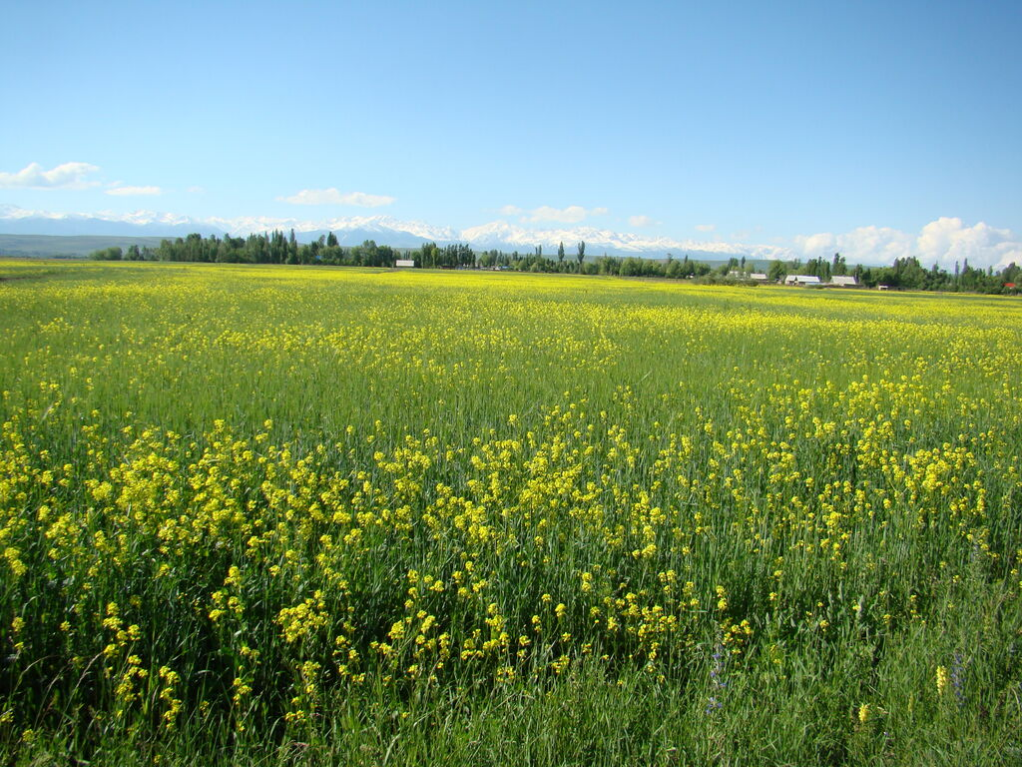
A wheat field with *Mutardaarvensis* near Karkara, Ysyk-Köl Region, Kyrgyzstan (photo by G. Lazkov, 15 June 2013).

According to the current record, *Mutardaarvensis* was primarily collected from argicultural lands (Fig. 18) in northern and eastern Kyrgyzstan. It was observed in fields at elevations from 750 to 2150 m a.s.l., with the common presence in the high-elevated Northern and Eastern Tian-Shan, contrary to the statement of Yarmolenko and Vasilczenko (1934) that the species prefers elevations below 1000 m a.s.l.

Its major presence in the country is registered on agricultural lands and ruderal places, including roadsides. The species was also not uncommonly found in natural, though usually disturbed habitats, like gravelly riversides, clayey deserts and meadows. The most remarkable case of its dispersal was observed in the western spurs of the Kök-Shaal Range, Eastern Tian-Shan, close to the Chinese border, where the species was found in 1939 in an uninhabited ravine at an elevation of ca. 2900 m a.s.l. This occurrence demonstrates its potential dispersal to the remote wilderness with horse fodder, mostly composed of grain, which has been commonly taken by shepherds and mounted guards to the mountains and deserts.

Besides the extensive weedy distribution, *Mutardaarvensis* has been cultivated for oil seed (Nikitina 1955). Its cultivation remains popular to date and accounts for a part of the alien occurrence, when the plants persist as ruderal or weedy leftovers of the former cultivation.

#### Ecology

The species strongly prefers open habitats on clayey and calcareous substrates (Fogg 1950). It survives droughts on cultivated land, but avoids naturally arid habitats.

#### Biology

Annual or winter annual with a slender taproot.

Cross- and self-pollination are combined to ensure stable seed set (Fogg 1950). The species has a high demand for water supply and may outcompete the crops for nutrients and water, thus reducing the yield severely or, in the worst cases, even completely (Zargar et al. 2021). Under favourable conditions, it may also outcompete other weeds (e.g. Alex (1970)). Seeds may remain viable when buried in soil and the plants re-appear when ploughing is resumed after at least 11 years (Chippindale and Milton 1934).

#### Notes

**Figure 19.**
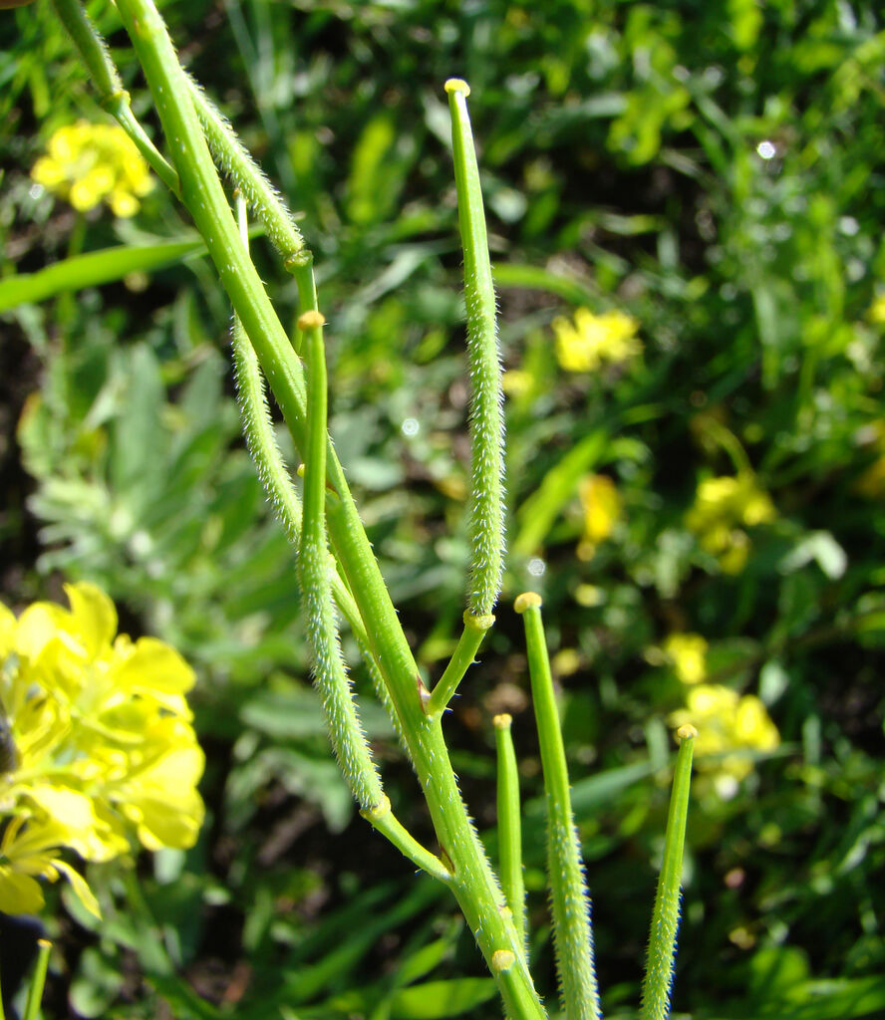
Mutardaarvensisvar.orientalis in a wheat field near Karkara, Ysyk-Köl Region, Kyrgyzstan (photo by G. Lazkov, 15 June 2013).

The species shows a high variability in some conspicuous morphological characters (Yarmolenko and Vasilczenko 1934). Its leaves may be entire, lyrate or lyrately dissected. Pods may be glabrous (Mutardaarvensisvar.arvensis) or hairy (Mutardaarvensisvar.orientalis (L.) Sennikov, comb. nov. Basionym: *Sinapisorientalis* L., Cent. Pl. I: 19 (1755); Fig. 19). This variability is largely infrapopulational and has no apparent geographical pattern.

#### Introduction to Kyrgyzstan

##### Period of introduction

Archaeophyte.

From the archaeophyte status of the species in Central Asia, we infer its first appearance in the territory of Kyrgyzstan in pre-historic times, through the agriculture in the Ferghana Depression.

##### Pathways of introduction

Transport - Contaminant: Seed contaminant. Escape from confinement: Agriculture.

*Mutardaarvensis* is a common contaminant of grain, fodder and ornamental seed and its primary alien occurrence originated from cultivated lands. Crop trade and accompanying losses in transportation and discharge places account for the ruderal occurrence of the species.

The species cultivation for oil seed contributes to its alien occurrence.

The secondary dispersal is limited and occurs primarily by winds and water currents along rivers.

##### Source of introduction

Iran, with the development of the Neolithic agriculture. Eastern Europe, with modern crop cultivation. Other sources are possible with imported grain.

##### Invasion status

Naturalised, not invasive. The species persists for uncertain time around the places of original introduction, intruding into neighbouring natural habitats, although its continuous presence in the territory may largely rely on the constant arrival of new diaspores through contaminated seed (like in the case of *Xanthiumstrumarium*: Sennikov and Lazkov (2021)).

##### Evidence of impact

Agriculture - major impact (noxious weed of all crops, in fields and gardens). Native ecosystems - moderate impact (occurring along streams and roadsides near populated places). Urban areas - moderate impact (ruderal occurrence).

##### Trend

Stable (observed).

### Rorippa austriaca

(Crantz) Besser, 1822

CFFEC363-EE58-57D7-B4E2-4210E2A15B4B

urn:lsid:ipni.org:names:288628-1

***Rorippaaustriaca*** (Crantz) Besser, Enum. Pl. Volh.: 103 (1822) — *Nasturtiumaustriacum* Crantz, Stirp. Austr. Fasc. 1: 15 (1762).

#### Distribution

##### Native distribution

Central, Eastern and South-Eastern Europe (including the zones of deciduous forest, forest steppe and steppe: Jonsell (1973)), Asia Minor, Caucasus, Western Siberia (adjacent to Europe), Central Asia (north-western Kazakhstan).

##### Secondary distribution

Western and Northern Europe, southern Siberia, Central Asia (mountains), East Asia, North America.

##### Distribution in Central Asia

**Figure 20.**
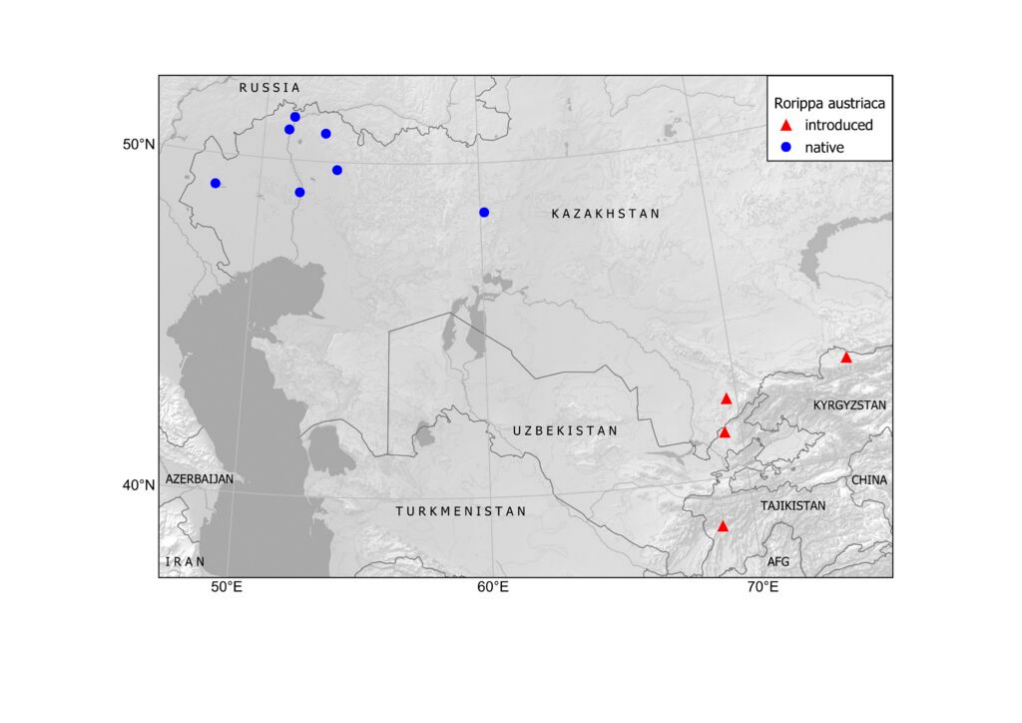
Native and alien occurrence of *Rorippaaustriaca* in Central Asia according to herbarium specimens examined and documented observations (iNaturalist 2024, Plantarium 2024).

Kazakhstan, Kyrgyzstan, Tajikistan, Uzbekistan. The distribution is mapped on the basis of herbarium specimens and published observations (Fig. 20).

In Central Asia, the species was first recorded as alien in Tajikistan, where a small, but established population was found in Dushanbe by Dorofeev (1984). The population, recorded in 1982 in the botanical garden of the Institute of Botany, Academy of Sciences, was found along an irrigation ditch; its age was estimated below 10 years.

In Kyrgyzstan, the species was found for the first and only time in Bishkek, as a weed of ornamental cultivation in 2009 (Lazkov et al. 2011).

In Uzbekistan, it was first found on a ruderal lawn in Tashkent in 1992 (German et al. 2013). Its continuous occurrence on city lawns has been subsequently confirmed in 2015 (Plantarium 2024).

**Figure 21.**
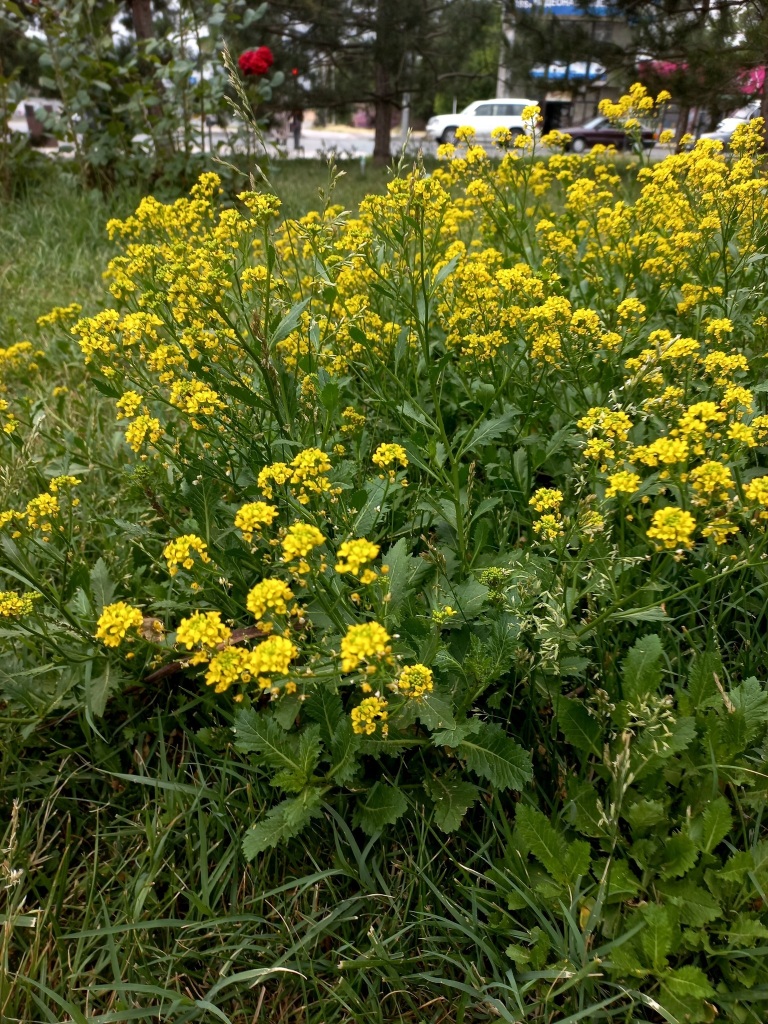
A stand of *Rorippaaustriaca* on a lawn in Şymkent, Kazakhstan (photo by A. Ebel, 15 May 2023). Source: https://www.inaturalist.org/observations/247918250 (iNaturalist 2024).

In Kazakhstan, the species is native in the steppe zone of the north-western regions of the country (Vasilieva 1961, Jonsell 1973); its easternmost limit coincides with the Mūğaljar Mts. (the southern extension of the Urals). As an alien, it was recorded once (Fig. 21) from a lawn in Şymkent Town in 2023 (Ebel et al. 2024).

##### Distribution in Kyrgyzstan

**Figure 22.**
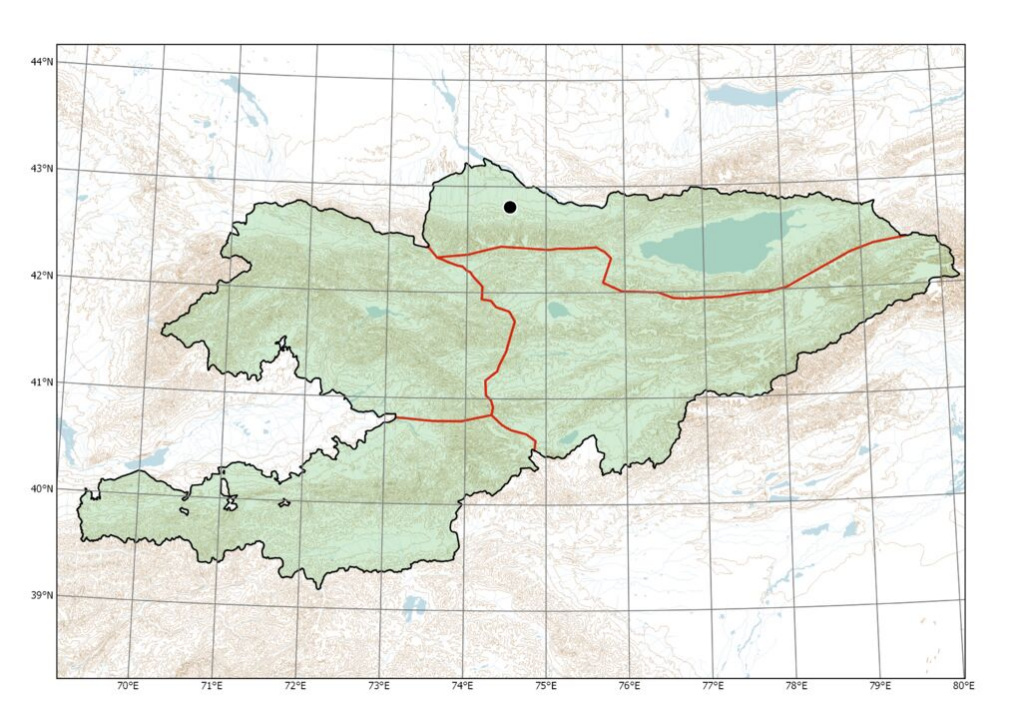
The record of *Rorippaaustriaca* in Kyrgyzstan.

Northern Tian-Shan (Fig. 22).

*Rorippaaustriaca* was found in Bishkek in 2009, recorded on a "lawn" (actually, among ornamental roses in block planting) in front of the main building of the Academy of Sciences in Bishkek (Lazkov et al. 2011).

#### Ecology

In the native distribution area, the species occurs in meadow and forb steppes, along watercourses or in temporarily inundated places. In the secondary distribution area, it is found on cultivated lands, in ruderal places and on roadsides, usually in connection with a good water supply.

#### Biology

Perennial, with short thickened rhizomes.

#### Introduction to Kyrgyzstan

##### Period of introduction

Neophyte.

As evident from the first records in Tajikistan and Uzbekistan, the introduction of *Rorippaaustriaca* to Central Asia has started in the late 1970s - early 1980s (Dorofeev 1984, German et al. 2013). The first observation of this species in Kyrgyzstan was dated by the post-Soviet period, by 2009 (Lazkov et al. 2011), but the actual introduction period may have started slightly earlier.

##### Pathways of introduction

Transport – Contaminant: Contaminant nursery material.

All observations in Central Asia have been made on cultivated lawns or flower beds (Dorofeev 1984, Lazkov et al. 2011, German et al. 2013, Ebel et al. 2024). These observations strongly indicate that the species was introduced with contaminated garden soil, transported with planting material and with contaminated seeds.

The species has a potential to spread along irrigation ditches, but no secondary dispersal has been observed in Kyrgyzstan.

##### Source of introduction

Introduced with ornamental plants via East European and then international nurseries.

##### Invasion status

Casual (ephemeral, extinct).

The only population registered in 2009 was ephemerous; it has been removed by management soon thereafter (Lazkov, pers. obs.). Further records are expected elsewhere and in the future, but not observed, likely due to the shortage of botanical observations.

##### Evidence of impact

Agriculture - minor impact (minor garden weed of infrequent occurrence). Native ecosystems - no impact (restricted to agricultural and urbanised areas). Urban areas - minor impact (rarely occurs in recreation places).

##### Trend

Unknown, but no apparent increase observed.

### Rorippa sylvestris

(L.) Besser, 1821

667E857D-B860-540A-A15E-0C7BBB37E959

urn:lsid:ipni.org:names:288692-1

***Rorippasylvestris*** (L.) Besser, Enum. Pl. Volh.: 27 (1821) — *Sisymbriumsylvestre* L., Sp. Pl.: 657 (1753).

#### Distribution

##### Native distribution

Europe (Northern Europe excepted), Asia Minor, Caucasus, Iran.

##### Secondary distribution

Northern Europe, Northern, Central and Eastern Asia, China, Northern Africa, Northern and Southern America. In its northern limit, the species reaches as far as the true Arctic in Iceland and Svalbard (Wasowicz et al. 2019). In Northern Asia, it is found across southern Siberia and in the Far East (Chepinoga et al. 2024). The species is highly invasive, at the active stage of expansion (Ebel 2016).

Jonsell (1968) has analysed the early records of *Rorippasylvestris* in North-Western Europe (from the Great Britain to Finland) and concluded that the oldest pathway of its dispersal was with ship ballast and boat traffic, but this pathway became obsolete already in the 19^th^ century and the early arrivals made no practical contribution to the recent invasion. The dramatic increase in the species distribution in Northern Europe was apparently connected with the intense gardening and transportation of the garden commodities, which has been especially noticeable since 1915 (Jonsell 1968).

The distribution, spread and invasion potential of *Rorippasylvestris* in Siberia, a large region adjacent to Central Asia, have been described in detail and documented by A. Ebel (Ebel 2000, Ebel 2016). He noted that the species was first collected as a weed in the botnical garden of the Tomsk University as early as 1925 and in many populated places in more recent times, thus indicating its arrival with gardening and garden commodities. Very remarkably, its numerous rather early records in 1933 and then in the 1960s-1990s are linked with the occurrence in natural wetland habitats, supporting the idea that the species may have been partly dispersed by waterfowl - at least spreading locally in this way, but likely also involving long-distance dispersal from man-disturbed habitats colonised by the species (Ebel 2000).

In North America, *Rorippasylvestris* has been common in the main area east of the Mississippi River in north-eastern United States and southern Canada (Rollins 1981), where it occurs in natural wet habitats along rivers and waterbodies and in gardens, with the first record published in 1818 (Stuckey 1966, Stuckey 1972). Similarly to the situation in Sweden (Jonsell 1968) and Siberia (Ebel 2000, Ebel 2016), the broad occurrence or even prevalence of the plants in natural habitats required the researcher to undertake special analysis to obtain evidence for an introduced status in the New World. The first species colonies appeared as a result of long-distance dispersal, mostly to large sea and river ports (Stuckey 1966). Groh (1936) noted that the species was spread mostly with nursery stock in the 20^th^ century, whereas Stuckey (1966) considered rivers as a major corridor in the secondary dispersal.

Scattered records of *Rorippasylvestris* at mills (Hanssen and Nordhagen 1930) indicate its potential ability to immigrate as grain contaminant. It is known to occur as impurity in grass seed (Salisbury 1961), thereby spreading to city lawns.

Both in its native and secondary distribution area, the species may abundantly invade cultivated lands on fertile soils and with sufficient water supply. Notably, its abundant occurrence in forest nurseries in Sweden (Bärring 2008) indicates a strong potential of the species to infest almost any kind of rooted saplings in commercial distribution, which played a major role in its secondary dispersal by man.

##### Distribution in Central Asia

**Figure 23.**
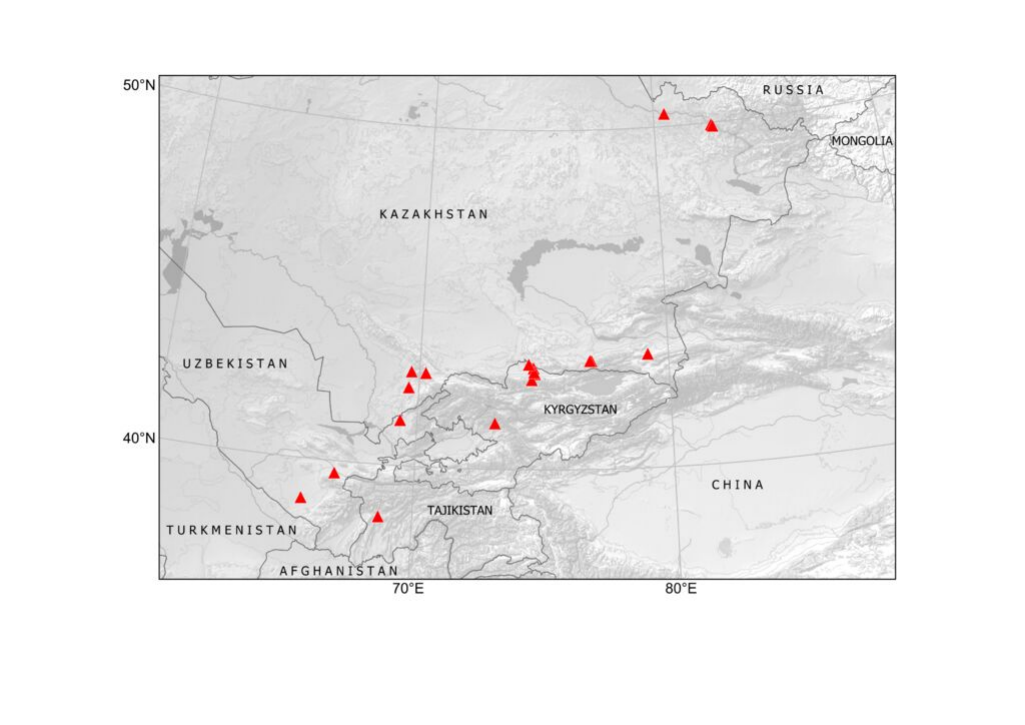
Distribution of *Rorippasylvestris* in Central Asia according to herbarium specimens and documented observations (iNaturalist 2024, Plantarium 2024).

Kazakhstan, Kyrgyzstan, Tajikistan, Uzbekistan (Fig. 23).

In Central Asia, *Rorippasylvestris* was first recorded in Uzbekistan, where it was found in a garden in Tashkent (Popov 1924). The documenting specimen was collected in 1919. Since then, the species has been repeatedly collected or observed as a weed or ruderal plant in Tashkent and keeps its presence as noted in recent local observations (e.g. Tillaev and Gaziev (2021)). It was also found in ruderal places in Samarqand in 1940 (Botschantzev and Vvedensky 1955) and Qarshi in 2024 (iNaturalist 2024).

The occurrences in Tashkent and Samarqand remained isolated in Central Asia until rather recent times (Shermatov 1974).

The first species record from Kyrgyzstan, noting its occurrence in Bishkek and vicinities (Jonsell 1973), has been commonly neglected in regional literature. The species distribution in the country is detailed for the first time in our present work.

In Tajikistan, the species has been found in 1982 in the botanical garden of the Academy of Sciences in Dushanbe, as a weed in ornamental cultivation and as a ruderal plant along irrigation ditches (Dorofeev 1984). It was subsequently observed in the city in 2017-2018 (Ebel et al. 2020).

In Kazakhstan, the species was first found along rivers within two large towns, Ust-Kamenogorsk in 1967 and 2002 (Ebel 2000, Ebel 2002) and Semipalatinsk in 2002 (Ebel 2002, Ebel and Ebel 2003).

##### Distribution in Kyrgyzstan

**Figure 24.**
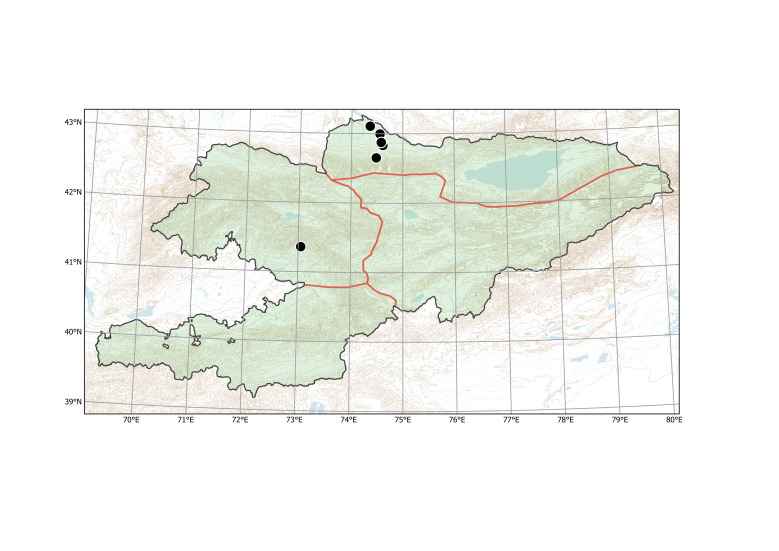
The currently known distribution of *Rorippasylvestris* in Kyrgyzstan according to herbarium specimens and documented observations.

Western Tian-Shan, Northern Tian-Shan (Fig. 24).

**Figure 25.**
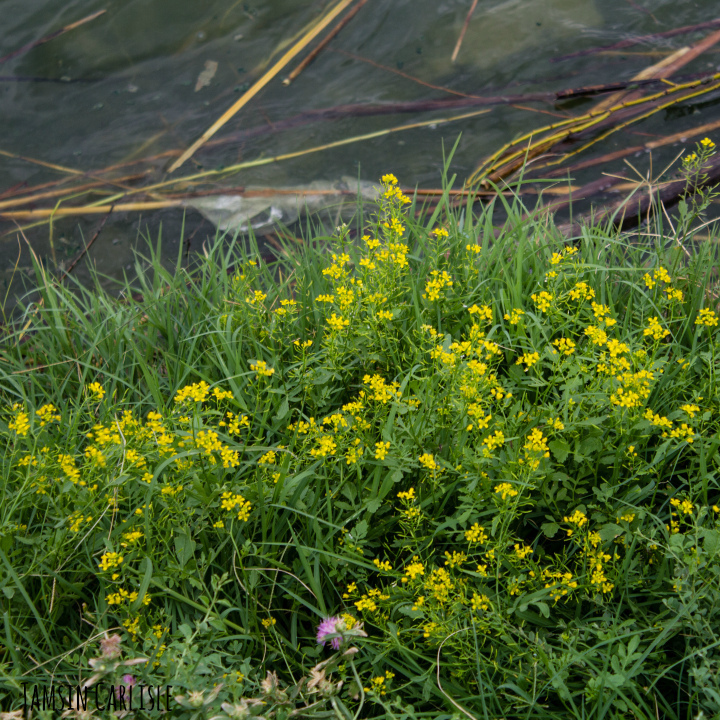
A stand of *Rorippasylvestris* along the Ala-Archa River above Bishkek City (photo by T. Carlisle, 23 July 2016). Source: https://www.inaturalist.org/observations/4131981 (iNaturalist 2024).

*Rorippasylvestris* was first collected in Kyrgyzstan in 1955, near Jangy-Jer Village in the Chüy Depression, north of Bishkek City. In the same area, the species was subsequently collected in Bishkek (city centre) and along the Ala-Archa River which streams from the mountains through Bishkek. To date, the plants have been found in several places along this river (Fig. 25), from the mountains to the lowland.

One more historical locality, also dated 1955, is known from the walnut forest area near Arstanbap Village. This locality has not been taken into account in the previous publications (Sennikov and Lazkov 2024a) and is formally reported as a new record here.

The localities are situated in the lowlands (700-800 m a.s.l.), foothills (1150 m a.s.l.) and the lower mountain belt (1300-1700 m a.s.l.).

#### Ecology

Moist meadows and riversides in the southern forest zone in the native distribution area. Riversides and inundated places, cultivated lands, lawns, roadsides and ruderal places in the secondary distribution area. Although the species is typically associated with wet habitats, it can endure extensive periods of drought; this ability allows it to colonise ruderal habitats (Salisbury 1961).

#### Biology

Perennial, rhizomatose.

Although the seed set may be limited because of a high level of self-sterility in monoclonous or genetically-poor introduced populations, it is compensated by the ability to actively disperse by root fragments (Stuckey 1966). The species has a strong ability to persist in cultivated lands due to rooting at nodes of its prostrate stems and regeneration from small fragments of rhizomes (Salisbury 1961).

#### Taxon discussion

Three ploidy levels (tetraploid, 2n = 32; pentaploid, 2n = 40; hexaploid, 2n = 48) are known in the secondary distribution area (Jonsell 1968). The Central Asian plants have not been karyologically tested.

Hybrids between *Rorippasylvestris* and *R.austriaca* are common when the species co-occur in their either native or secondary distribution (Bleeker 2007). Such hybrids are invasive in Europe and the hybridisation seems to facilitate their invasion (Bleeker 2003, Bleeker and Matthies 2005). The hybrid (*R.armoracioides* (Tausch) Fuss) has already been collected in Uzbekistan in 1954 (Jonsell 1973) and in north-eastern Kazakhstan in 2002 (Ebel 2002). It remains unknown in other countries of Central Asia.

#### Introduction to Kyrgyzstan

##### Period of introduction

Neophyte.

As the species is rather conspicuous, it is unlikely that its occurrence has long been neglected in the proximity to the capital and its botanical activities (cf. Stuckey (1966), Jonsell (1968), Ebel (2000)). With the first herbarium collections made in 1955, we may safely assume that the species was introduced in the late 1940s or early 1950s.

##### Pathways of introduction

Transport – Contaminant: Contaminant nursery material.

Since 1948, when the Communist Party of the USSR and the Soviet Government had announced that forest plantations are crucially important for agricultural productivity, territories of the Chüy Depression near Bishkek in Kyrgyzstan were involved in experimental planting of forest trees (Karafa-Korbut 1955). At that time period, planting trees became common also in the mountains, including nature reserves, as part of the programme for "improving" natural forests (e.g. Kotlar (1973)); we have observed remnants of such planting in many places in Kyrgyzstan, for example, the Sary-Chelek Nature Reserve (Lazkov and Ganybaeva 2021).

All occurrences of *Rorippasylvestris* in Kyrgyzstan are linked to forest plantations (the oldest records in the Chüy Depression, Ala-Archa River and a walnut forest area near Arstanbap Village) or ornamental cultivation (Bishkek City). We assume nursery transport to be responsible for all these introductions.

The secondary dispersal may occur by waterflow and by water birds along watercourses.

##### Source of introduction

Eastern Europe.

##### Invasion status

Naturalised.

The localities in the city may be ephemeral because of constant management, but the species is known as naturalised along the Ala-Archa River (recent observations in 2023: iNaturalist (2024)).

##### Evidence of impact

Agriculture - minor impact (the species is known as a weed of ornamental cultivation, although with low frequency and very limited distribution). Native ecosystems - minor impact (the species is locally spreading along natural and artificial watercourses). Urban areas - minor impact (the species occurs as a ruderal along pavements and irrigation ditches in populated places, with limited distribution).

##### Trend

Gradually increasing. The species is slowly expanding in native habitats and becoming more noticeable in the city.

### Sisymbrium irio

L., 1753

A2D5CA0C-6D0C-5505-86F2-B9D7FE6AE861

urn:lsid:ipni.org:names:237940-2

***Sisymbriumirio*** L., Sp. Pl. 2: 659 (1753).

#### Distribution

##### Native distribution

Zhou et al. (2001) considered the native distribution of *Sisymbriumirio* to cover the circum-Mediterranean area, Iran and Central Asia up to western China (Xinjiang). This opinion was followed by POWO (2024). However, the native distribution of the species is highly obscured by its extensive secondary dispersal.

Phylogenetically, *S.irio* is most closely related to *S.reboudianum* Verl. and *S.erysimoides* Desf., which are native to the Mediterranean, in general and North Africa, in particular (Žerdoner Čalasan et al. 2021). We circumscribe the native distribution of this species as covering the Mediterranean area only.

##### Secondary distribution

The species is widely naturalised in temperate Europe, South-Western Asia and Iran, Central Asia, North and South America, South Africa and Australia (POWO 2024). Two major areas of the species invasion are linked to crop production. In south-western USA and Mexico, an extensive area of the species naturalisation (Rollins 1980, Rollins 1981, Rollins and Al-Shehbaz 1986, Rollins 1993) is linked with a variety of crops, for example, alfalfa, maize and sugarbeet, but natural semi-deserts also appear to be heavily infested. In Australia, the species is widespread in all states, but the south-eastern territories are particularly affected by its invasion (Martín-Forés et al. 2023, GBIF 2024), with a major presence in oilseed crops (Salisbury et al. 2018).

In Asia, the species presence in Turkey seems to be minor (Hedge 1965) and therefore its native status is unlikely there. It is considered a common weed in Iran (Hedge 1968), where it may be an archaeophyte rather than native; similarly, it is an old anthropogenous incomer that has been established in the Indian subcontinent (Khoshoo 1966). Isolated localities of casual introduction are known in many countries in the Northern Hemisphere, as far northwards as Finland (Suominen 1979).

In Europe outside the Mediterranean, the species has spread northwards already during the 17^th^ through 19^th^ centuries (Salisbury 1961), but remains mostly casual or showing a negative trend (e.g. McCollin and Geraghty (2015), Pyšek et al. (2022)), although being capable for long-term persistence under favourable local conditions, such as in its legendary place of presence, London (Trimen and Thiselton-Dyer 1869, Clapham et al. 1989).

The species can be a noxious weed in some areas, as in the south-western United States (Rollins 1980), where it also invades abandoned lands in deserts (Burgess et al. 1991).

Trading commodities around the Globe continues to contribute to the further expansion of *S.irio*, as indicated by a report from Southern Korea, where the species was recently introduced through a trading port (Kim et al. 2021). A similar introduction has been recorded earlier in Japan (Kawamata et al. 2018).

##### Distribution in Central Asia

**Figure 26.**
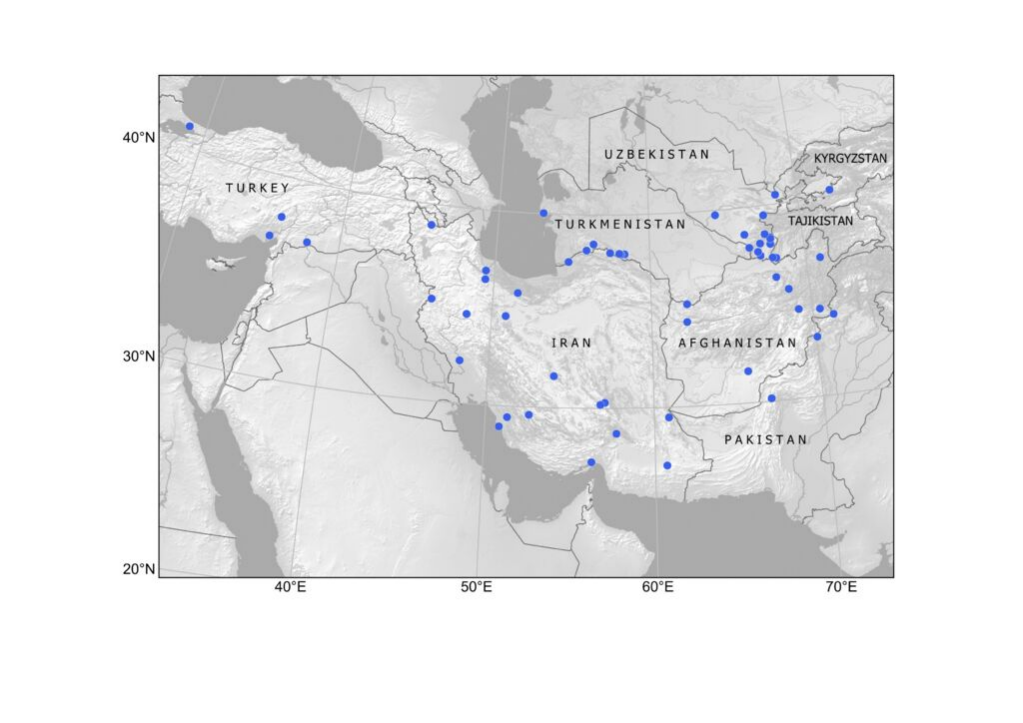
Native and alien (not distinguished) distribution of *Sisymbriumirio* in Central Asia and neighbouring countries (those named in the map).

The species is widely distributed in Central Asia and has been recorded from Kyrgyzstan (Bondarenko 1974), Tajikistan (Yunusov 1978), Turkmenistan (Vasilczenko 1948) and Uzbekistan (Botschantzev and Vvedensky 1955). We have mapped its occurrence in Central Asia and neighbouring countries (Fig. 26) on the basis of herbarium specimens at ALTB, LE, MW, P, TASH and literature data (Vasilczenko 1948, Yunusov 1978).

In Turkmenistan (Vasilczenko 1948), the species is relatively common in the mountainous area and has the same status as in Iran, i.e. likely an old archaeophyte, largely weedy. This part of the species distribution continuously extends from Iran and Afghanistan (Hedge 1968), except for the easternmost locality in the Kuhitang Mts. and the northernmost locality at the Caspian Sea, which are synanthropic.

**Figure 27.**
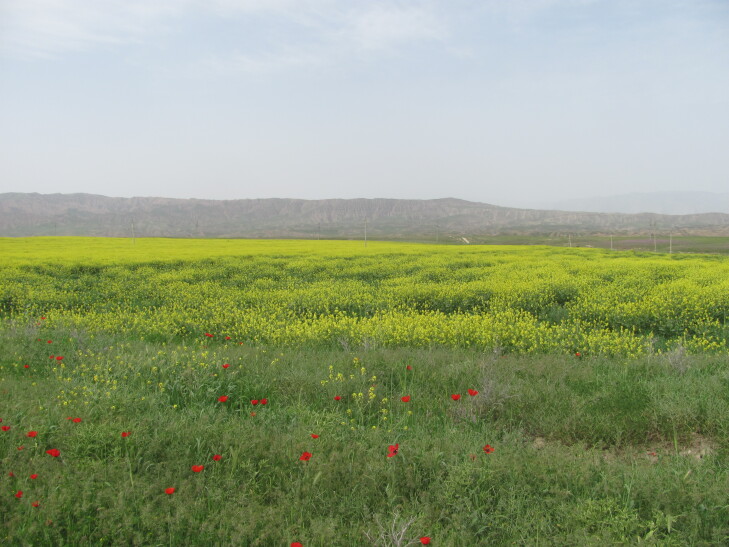
A fallow field south of Boysun Town in Uzbekistan, abundantly covered by *Sinapisarvensis*, also hosted plants of *Sisymbriumirio* (photo by D. German, 13 April 2023).

In Uzbekistan, the species is found in several localities in the southern part (Hissar Mts.), from which it has been repeatedly collected since the 19^th^ century; the oldest specimen from Sherabad Town was collected in 1881 (Franchet 1883). Due to their early collection dates, all its southern localities have originated from introductions prior to the Russian expansion to the region, i.e. from the sources in Afghanistan, from which grain have been supplied (Panin 2017) to compensate for food shortage in southern Hissar (Iskandarov 2012). Grain immigration pathway remains active in modern times, as evident from recent observations in Buxoro Town and on fields near Boysun Town (Fig. 27), although its modern source is currently unclear.

In Tajikistan, the species is known only from two published observations, which were based on specimens collected near the southern country border in the 1960s (Yunusov 1978). Recently, *S.irio* was also mapped as present in western Tajikistan, Zeravshan River Valley (Nowak and Nobis 2020), but the background collections have not been verified.

In Kyrgyzstan, the only occurrence was reported by Bondarenko (1974) and accepted in the latest checklist (Lazkov and Sultanova 2011, Lazkov and Sultanova 2014). The report was based on a single specimen: “Maidantal, ascending along Ak-Buura River from Kojo-Kelan to Kayyingdy Pass”, 05.1913, *Bronevski 154* (LE). This specimen was misidentified; it represents a flowering plant of *Sisymbriumloeselii* L. Moreover, Bondarenko (1974) misattributed its locality to the Fergana Range, thus misleading Sennikov and Tojibaev (2021) to include *S.irio* into their checklist of vascular plants of the Tian-Shan. The actual locality (Maidantal River, left tributary of Ak-Buura River) is situated in the Alay Range, which was explored by the botanical expedition of O. von Knorring in 1913 (von Knorring 1914).

Although we rejected the old record of *S.irio* in Kyrgyzstan, the species is reported from the country in the present contribution. There is another, obscure record of the species from rye fields in northern Kyrgyzstan, dated 5 May 2011 (Nowak et al. 2013), but we doubt its correct identity in the absence of publicly available herbarium specimens.

##### Distribution in Kyrgyzstan

Alay-Turkestan.

**Figure 28.**
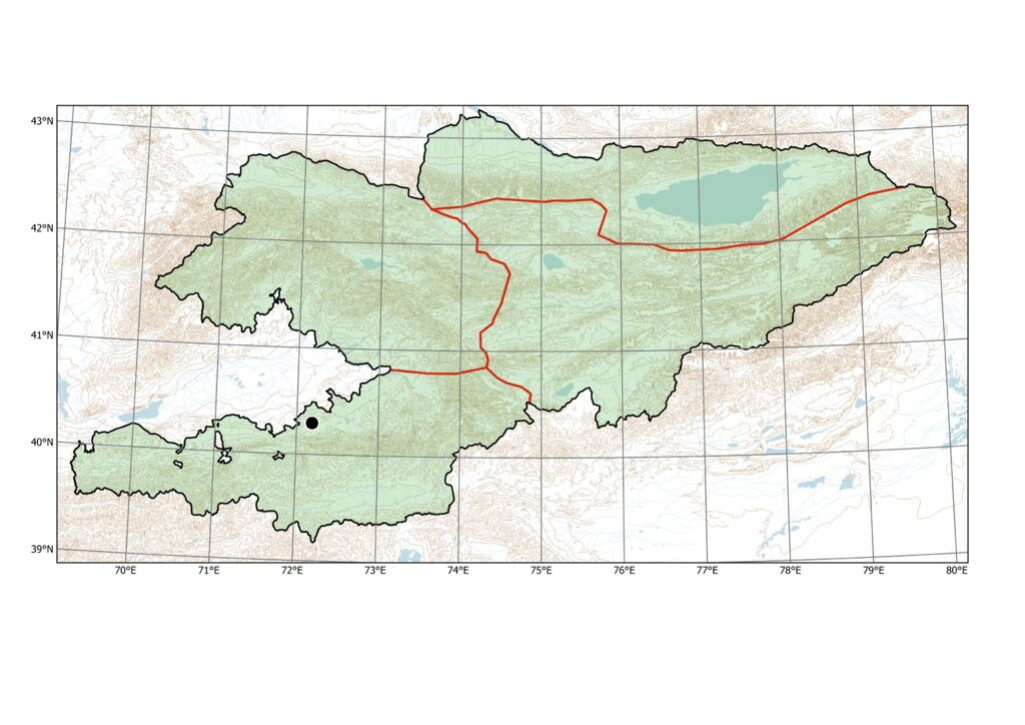
The confirmed alien record of *Sisymbriumirio* in Kyrgyzstan.

Two individuals of the species were observed and collected by D.German in 2024 on a roadside in the central part of Kerben Town, Jalal-Abad Region (Fig. 28). Since the previous species occurrence in Kyrgyzstan (Bondarenko 1974) has been rejected as based on a misidentification, this finding is a new country record.

#### Ecology

Semi-deserts in the native distribution area; cultivated and abandoned lands, a variety of disturbed grounds in the secondary distribution area.

#### Biology

*Sisymbriumirio* is an annual or over-wintering herb (Clapham et al. 1989).

The species is self-pollinating (Khoshoo 1966). It has a quick development cycle and is capable of producing numerous small seeds, which are easy to disperse. The species exists on several ploidy levels (diploid, triploid, tetraploid, hexaploid and octoploid) (Khoshoo 1955), which may facilitate its dispersal to different habitats and climate zones (Khoshoo 1965).

#### Notes

The species can be confused with *Sisymbriumloeselii*, which differs from *S.irio* by petals (5)6–8 mm long (vs. 2.5–3.5(4) mm long) and hispid (vs. soft) pubescence, when present (Yunusov 1978, Zhou et al. 2001, Al-Shehbaz 2015).

#### Introduction to Kyrgyzstan

##### Period of introduction

Neophyte.

The species has been recorded for the first time in 2024. Its first introduction may have gone unnoticed because of the ongoing flow of commodities from abroad, but the lack of earlier records suggests the rather recent arrival in the post-Soviet times.

##### Pathways of introduction

Transport - Contaminant: Seed contaminant.

Three major pathways of introduction are known for the species. In Europe, it was most frequently found as a grain impurity (Suominen 1979, Rich 1991) or in wool waste (Pyšek 2005, Shimwell 2006). A recent, but seemingly minor means of introduction is soil contamination; the plant was found in containers in topsoil with imported ornamental plants in the Mediterranean trade (Hoste and Verloove 2010). In particular, this pathway was assumed in the latest report of *S.irio* from Siberia (Bolbotov et al. 2024).

In Kyrgyzstan, we can safely assume the species immigration with grain import. Further dispersal has not been observed.

##### Source of introduction

Probably Uzbekistan, due to the trade within Central Asia.

As the plants were observed growing on a roadside without any direct connection with the source, their link with a particular commodity is uncertain. *Sisymbriumirio* is commonly introduced with wheat (e.g. Suominen (1979)), which may also be the case here, but its origin is uncertain. Wheat import has covered a third of the total consumption in Kyrgyzstan and this commodity has been recently imported mostly from Kazakhstan (Zorya et al. 2020) and, to a minor extent, from Russia, whereas *S.irio* is only a rare railway alien in Russia (Dorofeev 2002) and has never been reported from Kazakhstan (Abdulina 1999), so that the species immigration with grain from these countries is impossible. However, according to our observations, the species is present as a weed on wheat fields in Uzbekistan, from which some irregular import occurs, especially in border regions. Uzbekistan could be a likely origin of seed propagules of *S.irio* in Kyrgyzstan.

##### Invasion status

Casual.

The species persistence has not been observed. Its record in a populated place suggests casual introduction, with low chances for survival in this particular locality. However, future naturalisation is not excuded as the species exhibits a high capacity for naturalisation in semi-deserts (Rollins 1980, Rollins 1993) and its presence in Uzbekistan seems to be persistent.

##### Evidence of impact

Agriculture - no impact (so far, not recorded on fields, although recent surveys are lacking). Native ecosystems - no impact (not found outside populated places). Urban areas - minor impact (casual occurrence as a ruderal plant).

##### Trend

Increasing (inferred).

The high level of wheat import to Kyrgyzstan, which mostly occurs within the Central Asian region (Zorya et al. 2020), explains the recent introduction of *S.irio* to the country and demonstrates its potential for further spread with imported commodities.

## Discussion

Our analysis of the complete historical data on the distribution of eight species of Brassicaceae in Central Asia has demonstrated certain trends and tendencies which are important for understanding the timing and origin of plant invasions in Central Asia. This information provides a further detalisation of the knowledge obtained in our previous research (Sennikov and Lazkov 2021, Sennikov and Lazkov 2022).

The extent of naturalisation of alien plant species in northern Kazakhstan is generally underestimated. This is especially true in respect of cultivated (edible or ornamental) plants, which were typically not included into botanical manuals and, therefore, not collected and monitored by professional botanists (e.g. *Armoraciarusticana*). The plants have been originally introduced by colonists during the 19^th^ century from the central and southern European parts of the Russian Empire and repeatedly transported with new immigrations and supplies in the Soviet times. Such plants not only colonised fallow fields and ruderal habitats, but also became established in natural habitats; they first appeared along riversides within populated places, possibly originating from garden waste (Rusterholz et al. 2012) and then became dispersed by water currents along the rivers.

Cultivated plants may arrive in the territory through widely different pathways even in a short historical period and good care is needed to uncover and separate the means of introduction. A good example from the present contribution is *Crambeorientalis*, whose alien occurrence originated through experimental field cultivation and ornamental garden use, but also unaided by natural means pertaining to its tumbleweed life-form from the territory previously colonised by the species. The sporadic occurrence of the species, combined with the historical botanical records, still allows recognition of its original introduction areas and pathways.

The immigration and spread of agricultural weeds in Central Asia was different in the steppes of Kazakhstan, with their traditionally nomadic population and in the largely mountainous territories of the former khanates or emirates (Buxoro, Xorazm and Qoqon) with their settled population and long-developed agriculture. The steppes have been settled by Russian colonists and influenced by the Russian grain import for some 200 years, in a sequence of waves: Cossack settlement (before the 1880s) and mass colonisation from Eastern Europe (since the 1880s), followed by the establishment of collective farms (in 1928-1932), machinery-based agriculture (in 1936-1940) and Virgin Land campaign (in the 1960s); the latter led to the introduction of exotic alien plants, for example, *Xanthiumorientale* (Sennikov and Lazkov 2021). Agricultural valleys in the mountains developed the tradition of grain cultivation since the Neolithic period, which has arrived from Iran and remained connected with that country through the trade and cultural ties; this activity is responsible for the immigration of numerous agricultural weeds from the south-west. With the Russian conquest of the three khanates in the 1860s-1880s, "Russian" (including Ukrainian and Caucasian) settlers have contributed to the flora by the first transportation of weeds and cultivated plants. This first wave of introduction was subsequently supplemented with the Soviet reorganisation and development of agriculture, i.e. the industrialisation and collective farming (during the first and second five-year plans, 1928-1938), as part of the common history of the USSR.

The immigration of weedy plants with contaminated grain from overseas has also occurred in a sequence of major events. Although annual grain crop yields fluctuated widely (Gidadhubli 1973), the Russian Empire and then its successor, the USSR, was self-sufficient in bread supply and remained as such until the catastrophic decline of its crop production in 1963 (Brown 1982). Some amount of wheat grain is known to have been received when the German invasion devastated the European part of the USSR during the Second World War and caused the national bread production to move to the Asian parts of the country; at that time, the shortage of wheat grain was partly compensated by the lend-lease import from the USA (Ryzhkov 2012), but no records of alien plants have been linked to this particular import. In general, the Soviet grain import was minor and casual in 1950s (Moravcik 1978), although the use of American seed was inferred from the emergence of new exotic weeds in Kazakhstan at the time of the Virgin Lands campaign of 1954-1963 (Sennikov and Lazkov 2021). The grain import remained extensive from 1963 until the end of the Soviet times (Tulpule et al. 1992), thus affecting Central Asia together with other parts of the USSR and bringing new exotic weeds as seed contaminants (Sennikov and Lazkov 2021, Sennikov and Lazkov 2022).

These historical observations on the pace of the introduction of alien plants in Central Asia, coupled with its gradual colonisation in the Russian Empire and the development of agriculture in the USSR, agree with the globally traced botanical legacy of other great European empires (Lenzner et al. 2022).

In the independence period, grain import to the arid mountain areas of Central Asia has gradually shifted from Russia and northern Kazakhstan to the extensive involvement of other countries, despite a higher level of associated costs. The use of fodder grain imported from remote countries is responsible for emerging ruderal occurrences of new exotic weeds, which can be seen especialy in the latest years.

In general, major historical events are closely connected with the introduction and further dispersal of alien plants in Central Asia, as shown by the botanical records uncovered and interpreted in the present contribution. The reasonable density of historical records, caused by the high level of floristic activities in the Russian Empire and the USSR, allows for distinguishing between native and alien occurrences of the same species and for tracing historical events leading to the plant invasions in the territory, thus providing the background for informed decisions regarding the causes of arriving and spreading of unwanted plants in Central Asia.

The currently available distributional records of alien plants in Central Asia are mostly historical, with rather few additions that mostly focused on exotic introductions; although these data provide a good hint on the history of introduction, they may not demonstrate the actual occurrence of common weeds. Modern surveys are urgently needed to reveal the situation because this data deficiency may obscure and hide even major invasions, as occurred with the late detection of *Bidensfrondosa* in Central Asia (Sennikov and Lazkov 2022).

## Supplementary Material

XML Treatment for Armoracia rusticana

XML Treatment for Crambe orientalis

XML Treatment for Hesperis pycnotricha

XML Treatment for Hirschfeldia incana

XML Treatment for Mutarda arvensis

XML Treatment for Rorippa austriaca

XML Treatment for Rorippa sylvestris

XML Treatment for Sisymbrium irio

3106A4A2-10C4-526F-911E-06B9C6BA566B 10.3897/BDJ.13.e145624.suppl1 Supplementary material 1 Distributional dataset for alien species of Brassicaceae in Central Asia (excluding Kyrgyzstan) Data type occurrences Brief description An occurrence dataset for the alien species of Brassicaceae in Central Asia, which are present in Kyrgyzstan and occur in Kazakhstan, Tajikistan, Turkmenistan and Uzbekistan. The dataset is organised in the DarwinCore format. It is based on plant occurrences which are traced from herbarium collections (AA, ALTB, FRU, LE, MW, P, TASH), documented observations (Plantarium) and literature (Flora Iranica, Flora of Tajikistan, Flora of Turkey, Flora of Turkmenistan). File: oo_1208295.csv https://binary.pensoft.net/file/1208295 Sennikov, A.N.; German, D.A.

174EC2C7-4101-51C7-A8CD-A52B4465407C 10.3897/BDJ.13.e145624.suppl2 Supplementary material 2 Distributional dataset for alien species of Brassicaceae in Kyrgyzstan Data type occurrences Brief description An occurrence dataset for the alien species of Brassicaceae in Kyrgyzstan. The dataset is organised in the DarwinCore format. It is based on plant occurrences which are traced from herbarium collections (ALTB, FRU, LE, MW, TASH) and documented observations. File: oo_1208290.csv https://binary.pensoft.net/file/1208290 Sennikov, A.N.; Lazkov, G.A.; German, D.A.
